# Preparation and Efficacy Evaluation of Antihyperuricemic Peptides from Marine Sources

**DOI:** 10.3390/nu16244301

**Published:** 2024-12-12

**Authors:** Kun Qiao, Qiongmei Huang, Tongtong Sun, Bei Chen, Wenmei Huang, Yongchang Su, Hetong Lin, Zhiyu Liu

**Affiliations:** 1 Engineering Research Center of Fujian and Taiwan Characteristic Marine Food Processing and Nutrition and Health, Ministry of Education, College of Food Science, Fujian Agriculture and Forestry University, Fuzhou 350002, China; qiaokun@xmu.edu.cn (K.Q.); 1220919043@fafu.edu.cn (Q.H.); hetonglin@163.com (H.L.); 2 Key Laboratory of Cultivation and High-Value Utilization of Marine Organisms in Fujian Province, National and Local Joint Engineering Research Center for Marine Biological Seed Industry Technology, Fisheries Research Institute of Fujian, Xiamen 361013, China; beifjfri@foxmail.com (B.C.); suyongchang@126.com (Y.S.); 3 College of Food Sciences & Technology, Shanghai Ocean University, Shanghai 201306, China; M220300937@st.shou.edu.cn; 4 Xiamen Daozhiyuan Biological Technology Co., Ltd., Xiamen 361024, China; daozhiyuan188@163.com

**Keywords:** hyperuricemia, marine bioactive peptides, xanthine oxidase, uric acid

## Abstract

Marine-derived foods, often called blue foods, are promising sustainable alternatives to conventional food sources owing to their abundant amino acids and high protein content. Current treatments for hyperuricemia, a chronic condition attributed to purine metabolism disorders, are associated with various side effects. Novel peptide xanthine oxidase inhibitors have been discovered in the hydrolyzed products of marine fish and invertebrate proteins, which have demonstrated promising therapeutic potential by reducing uric acid levels in vitro and in vivo. This review explores the potential therapeutic effects of xanthine oxidase inhibitors derived from marine fish and invertebrates, summarizes the methods for extracting bioactive peptides from marine organisms, and emphasizes the impact of different proteases on the structure–activity relationship of bioactive peptides. The hypouricemic effects of these bioactive peptides warrant further verification. There is consensus on the in vitro chemical methods used to verify the xanthine oxidase inhibitory effects of these peptides. Considering several cell and animal model development strategies, this review summarizes several highly recognized modeling methods, proposes strategies to improve the bioavailability of bioactive peptides, and advocates for a diversified evaluation system. Although the screening and evaluation methods for antihyperuricemic peptides have been shown to be feasible across numerous studies, they are not optimal. This review examines the deficiencies in bioavailability, synthesis efficiency, and evaluation mechanisms in terms of their future development and proposes potential solutions to address these issues. This review provides a novel perspective for the exploration and application of marine-derived hypouricemic bioactive peptides.

## 1. Introduction

Earth’s oceans cover 71% of the planet’s surface and represent a vital ecosystem, offering evolutionary advantages for marine flora, fauna, and microorganisms [1,2]. The global demand for aquatic food has increased exponentially, with projections indicating a doubling by 2050 [3]. Similarly, the demand for high-quality marine proteins has greatly increased [4]. Animal proteins are highly valued for their high bioavailability, biological value, and protein digestibility-corrected amino acid score [5]. There is evidence of positive correlations between the consumption of marine fish and human health [6]. Furthermore, the amino acid profiles of marine animal proteins are highly complementary to the amino acid requirements of the human body, resulting in enhanced bioavailability [7].

In recent decades, shifts in lifestyle and dietary patterns have resulted in a marked increase in the prevalence of hyperuricemia, a chronic condition attributed to purine metabolism disorder and the second most prevalent metabolic disorder after diabetes. This trend is evident in younger individuals [8].

**Figure 1:**
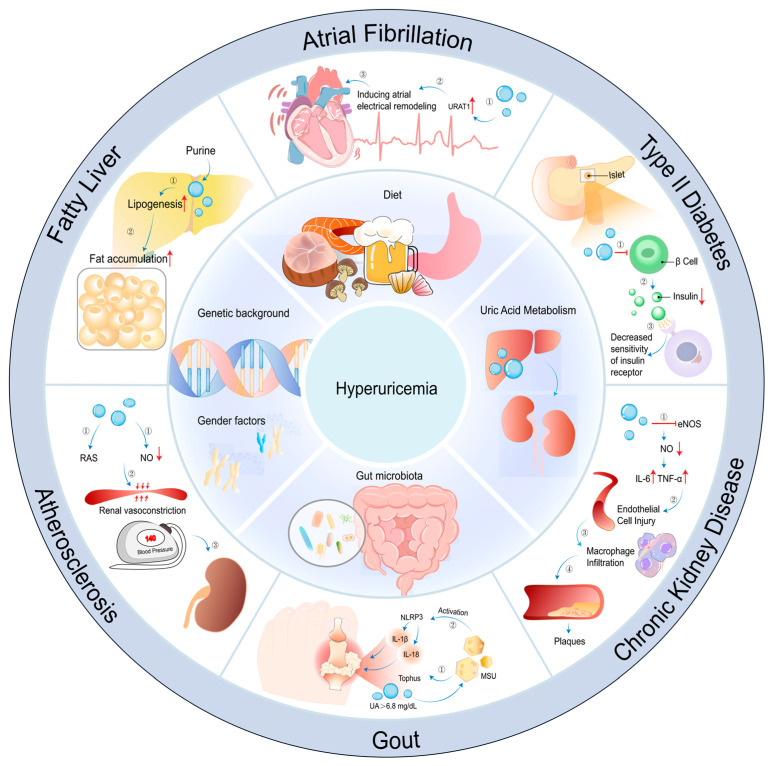
Main causes and complications of hyperuricemia.

Several factors have been identified as common risk factors for hyperuricemia, including the excessive intake of purine-rich diets, sex, age, genetic background, and abnormal gut flora [9,10,11,12] (Figure 1). The primary cause of hyperuricemia is the overproduction or underexcretion of uric acid. Strategies for managing hyperuricemia primarily encompass dietary management, regular monitoring, and pharmacological interventions, which provide the fundamental basis for restoring and maintaining long-term uric acid homeostasis. The pharmaceutical agents most commonly utilized in clinical practice are allopurinol and febuxostat. The advantage of these drugs lies in their ability to block the binding of xanthine oxidase with its substrates, thereby directly reducing uric acid production through the synthetic pathway. While these medications are effective, their use, either as monotherapy or in combination with other drugs, is associated with adverse effects [13,14,15,16,17,18]. The greatest challenge currently faced in the treatment of hyperuricemia is the mismatch between the side effects of medications and their therapeutic benefits. Thus, the search for alternative drugs that are suitable for long-term use and have a higher safety profile is crucial in the management of hyperuricemia, and food-derived active substances with potential hypouricemic effects have attracted the attention of researchers. The hypouricemic activity of food is closely correlated with its constituent ingredients [19]. Marine organisms are a rich source of bioactive substances, some of which have demonstrated significant antihyperuricemic effects in both in vitro and in vivo models [20]. These effects suggest the potential of these substances to inhibit xanthine oxidase activity and reduce uric acid levels. Consequently, bioactive peptides from marine organisms are considered to be potential xanthine oxidase inhibitors.

Bioactive peptides are small protein fragments comprising 2–20 amino acids [21] that exert beneficial biological effects following their release from protein structures. The applications of bioactive peptides are diverse, encompassing functional foods and beverages, dietary supplements, pharmaceuticals, cosmeceuticals, and animal feeds. In 2023, the global bioactive peptide market (excluding peptide drugs) was valued at 4.961 billion USD, representing an increase of 101 million USD compared to 2020. Projections indicate that this market will reach 10.711 billion USD by 2030. This trend reflects the increasing importance of bioactive peptides. The BIO-PEP-UWM database catalogs over 4800 bioactive peptides with a range of biological activities, including antioxidant, antihypertensive, antimicrobial, anti-inflammatory, and antidiabetic properties, which provides a valuable resource for the development of novel health products.

Terrestrial animal-derived proteins are the primary source of proteins for humans. However, with the increasing global population and limited food resources, there has been growing interest in alternative sources of protein, such as plant-based, insect-based, and aquatic resources, as outlined in reference [22]. Peptides derived from terrestrial animals have been demonstrated to possess notable pharmacological properties [23]. The angiotensin-converting enzyme (ACE) inhibitory peptides Val-Pro-Pro (VPP) and Ile-Pro-Pro (IPP) and the antihypertensive peptide Ile-Arg-Trp (IRW), derived from animal proteins, have been extracted and used on a commercial scale [24,25]. However, the emergence of diseases in terrestrial animals and avian influenza has raised concerns regarding the safety of products derived from terrestrial animal proteins. Furthermore, religious and customary practices may prohibit the use of protein-derived products derived from terrestrial animals [26]. The diversity of marine species and their extreme growth conditions may cause the production of protein precursors and secondary metabolites that are not found in terrestrial organisms [7], generating considerable research interest [27]. The abundance of resources and safety of proteins derived from aquatic organisms make them a highly promising source of protein derivatives [28]. YSK (Shizuoka Prefecture, Japan), has launched a dipeptide hypouricemic product, the primary components of which are alanylglutamine (7.3%) and glycylglutamine (90.9%), extracted from the skipjack muscle. The product demonstrated the potential for the extraction of peptides with antihyperuricemic properties from marine animal proteins.

Published articles on substances with antihyperuricemic activity have predominantly focused on terrestrial-derived foods and traditional Chinese medicinal herbs. In contrast, the development of marine-derived antihyperuricemic peptides is a relatively novel area that has been less frequently discussed. This review examines the synthesis and metabolic pathways of uric acid as well as pharmacological treatments for hyperuricemia. It also discusses the characterization, efficacy evaluation, mechanism of action, and structure–activity relationships of antihyperuricemic peptides derived from various marine animals. This review also provides a summary of the specific methods involved in the synthesis and efficacy evaluation of antihyperuricemic peptides, integrating computer simulation techniques and software that have practical application value in this process. This review elucidates the potential applications and sustainable development of marine-derived natural proteins in the context of hyperuricemia, aiming to broaden and deepen the exploration of oceanic bioactive substances, offering fresh insights for their development.

## 2. Methods

To systematically retrieve relevant references on hyperuricemia, marine organisms, and peptides, we performed a comprehensive search in the Web of Science and PubMed electronic databases, focusing on English-language publications from 2014 onwards, as this period marks a significant surge in research and investigations concerning antihyperuricemic peptides. We strategically applied Boolean operators, including AND/OR, to effectively intersect and broaden our search across various keywords. The citations and abstracts obtained from these searches were subsequently imported into NoteExpress software 4.0 for deduplication, ensuring a refined and non-redundant collection of studies for our review.

## 3. Uric Acid Overview

Uric acid, or 2,6,8-trioxypurine, is a weak organic acid with a pH of 5.8 and a chemical formula of C_5_H_4_N_4_O_3_ [29]. At physiological pH of 7.4 and 37 °C, most uric acid exists in an ionized state as slightly soluble urate salts that circulate in the blood and synovial fluid [30]. The typical reference range for uric acid in the blood of females is 1.5–6 mg/dL, whereas that of males is 2.5–7.0 mg/dL. The mean solubility limit for uric acid is 6.8 mg/dL.

When the concentration of urate salts in the serum exceeds the saturation threshold, it readily precipitates as monosodium urate (MSU) [31]. This process involves interactions between urate molecules, sodium ions, and water molecules and is influenced by multiple factors [14,15,16,17,18,19]. The serum uric acid concentration is a factor that affects the risk of urate salt crystallization and nucleation. MSU primarily induces local and tissue damage by triggering the synthesis of inflammatory factors and promoting the phagocytic response of phagocytic cells, which induces other complications, such as atherosclerosis [32,33], chronic kidney disease [34,35], hypertension [36,37,38], and obesity [39,40].

**Figure 2:**
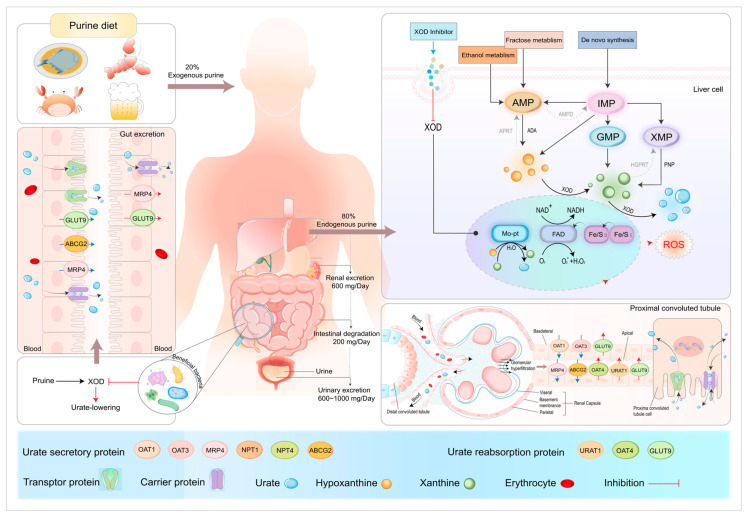
Schematic illustration of uric acid synthesis and transport.

Approximately 20% of exogenous uric acid in humans is derived from the breakdown of nucleic acids in food, whereas the remaining 80% is produced endogenously via purine metabolism [41]. We have depicted the primary pathways of uric acid synthesis and metabolism in Figure 2. The liver is the primary site for uric acid synthesis, where purine nucleotides, mainly adenine and guanine, are catalyzed by xanthine oxidase (XOD) and adenosine deaminase (ADA) to produce inosinic acid, inosine, and hypoxanthine. These enzymes are key enzymes involved in this process [42]. ADA catalyzes the metabolism of inosine monophosphate (IMP) to inosine, which is further degraded to hypoxanthine by purine nucleoside phosphorylase (PNP) [43]. In contrast, XOD, a form of xanthine dehydrogenase (XDH), is a substrate-nonspecific hydroxylase that primarily functions to irreversibly convert hypoxanthine and xanthine to uric acid while reducing NAD^+^ or O_2_. The molybdenum cofactor at the active site of XOD represents the primary binding site for the purine substrates. Therefore, it is essential to assess the hypouricemic effects of these medications in terms of their impact on the enzymatic activities of XOD and ADA.

Approximately 70% of uric acid is excreted through the kidneys [44], a process mediated by several urate transport proteins including organic anion transporter 1 (OAT1), organic anion transporter 3 (OAT3), ATP-binding cassette subfamily G member 2 (ABCG2), glucose transporter 9 (GLUT9), and urate transporter 1 (URAT1) [31]. Urate transport proteins facilitate the secretion and reabsorption of uric acid. A reduction in uric acid excretion is regarded as the primary underlying cause of most cases of hyperuricemia [45]. Due to a genetic mutation in the uric acid oxidase gene, humans cannot synthesize uricase, an enzyme responsible for the breakdown of uric acid into the more soluble compound allantoin [46]. This genetic variation results in human serum uric acid levels approximately 10% higher than those observed in other mammals [47].

## 4. Hyperuricemia Pharmacotherapy

Hyperuricemia can be attributed either to the excess production or inadequate excretion of uric acid [48]. In adults, the condition is defined as a serum uric acid concentration exceeding 7.0 mg/dL for males and 6.0 mg/dL for females. The pharmacological treatment of hyperuricemia is primarily categorized into three groups: xanthine oxidase inhibitors (XOIs), urate-lowering therapies that target urate anion exchange transporters, and uricase-like agents.

According to the 2012 American College of Rheumatology guidelines, allopurinol and febuxostat are recommended as the initial therapeutic options for symptomatic hyperuricemia [49]. Allopurinol competitively inhibits the catalytic activity of the enzyme by binding to the reduced form of xanthine oxidase, forming an inert complex. However, as a purine analog, allopurinol may disrupt the normal purine metabolism. Approximately 70% of allopurinol is metabolized to oxypurinol, which increases the renal excretion burden. A cross-sectional study conducted in Japan revealed that high doses of allopurinol may diminish the response to erythropoiesis-stimulating agents in patients with chronic kidney disease [50]. Febuxostat, a non-competitive inhibitor, differs from allopurinol because it is primarily metabolized by the liver. Although it does not contribute to the renal metabolic burden, it may cause liver function abnormalities [51]. Benzbromarone, an effective URAT1 inhibitor [52], is an option for patients who are intolerant to XOIs. However, its hepatotoxicity and low URAT1 selectivity have restricted its clinical application [53]. Uricase-like agents, including rasburicase and pegloticase, facilitate the oxidation of uric acid to allantoin. These pharmaceutical agents are costly, have a short half-life, and can readily induce hypersensitivity reactions [54].

Although these medications are effective in the treatment of hyperuricemia, they are associated with a range of adverse effects. Previous studies and clinical trials have demonstrated that the combined use of certain drugs is more effective and safer than monotherapy. However, some limitations remain, including the risk of rebound hyperuricemia after treatment discontinuation and poor medication adherence. These factors are not conducive to the effective long-term management of uric acid levels [55].

In accordance with international guidelines, individuals with elevated uric acid levels and asymptomatic hyperuricemia are initially advised to manage their uric acid levels through dietary modifications. However, dietary management can cause adverse effects. While a low-purine diet can mitigate the risk of hyperuricemia by reducing purine intake, it concomitantly diminishes the intake of certain nutrients required by the body. Mao et al. [56] analyzed four dietary patterns among Chinese adults. These findings indicated that diets low in egg and fish protein, as well as diets high in meat and fish protein, may be associated with increased blood uric acid levels. Individuals with elevated blood uric acid levels require rigorous dietary management, which frequently entails heavy financial investment in lifestyle modification.

Recently, bioactive peptides have garnered attention owing to their potential to promote health. Compared with small molecules, bioactive peptides possess several advantageous properties, including facile absorption, minimal off-target toxicity, high activity, and stability [57,58,59]. These characteristics render bioactive peptides as promising candidates for the prevention and treatment of hyperuricemia. It is noteworthy that bioactive peptides derived from natural sources represent a novel strategy for the daily management of hyperuricemia, thereby alleviating the burden of dietary restrictions. By targeting pivotal pathways involved in uric acid production and metabolism, these peptides can facilitate the regulation of blood uric acid levels, offering a prospective treatment option for patients and enhancing their quality of life. As research progresses, bioactive peptides have the potential to become indispensable components of hyperuricemia management.

## 5. Classification of Marine Source XOD Inhibitory Peptides

Aquatic organisms are a rich source of amino acids, and hydrolysis processing can yield bioactive peptides with small molecular weights and high bioavailabilities. These exogenous peptides obtained through specific processes from food sources are called food-bioactive peptides (FBPs) [60].

Besides providing nutritional value, FBPs also perform physiological regulatory functions [61]. To avoid confusion with protein hydrolysates derived from food sources, the identified peptide sequences will be categorized as FBPs in the following discussion. Table 1 lists the pretreatment methods for marine vertebrate and invertebrate proteins, methods for preparing protein hydrolysate, and the previously identified peptide sequences with XOD inhibitory activity.

Marine fish, particularly tuna, have emerged as valuable sources of XOD inhibitory peptides (Table 1). The tuna muscle tissue contains creatine and imidazole compounds. A recent randomized controlled trial compared the effects of placebo with those of tuna extract on uric acid levels and found that oral intake of tuna extract may reduce blood uric acid levels. Specifically, the authors proposed that tuna extract promotes the expression of hypoxanthine–guanine phosphoribosyl transferase, reducing the precursors of uric acid and simultaneously enhancing the activity of lactate dehydrogenase, promoting the reabsorption and excretion of uric acid. Furthermore, creatine action in the bloodstream helps maintain a stable pH, which increases uric acid solubility [62].

The results of our literature review indicate that, compared to XOD inhibitory peptides extracted from marine fish, those derived from marine invertebrates remain limited. In a comparative analysis of the XOD inhibitory effects, Huang et al. [63] evaluated the inhibitory capacity of 400 dipeptides extracted from tilapia, white shrimp, and imitation bonito. These findings indicated that the dipeptides derived from tilapia demonstrated the most pronounced XOD inhibitory activity. However, it is erroneous to assume that marine fish are inherently superior to marine invertebrates as extraction materials. To illustrate, the hexapeptide ALSGSW, extracted from oysters, exhibits an IC_50_ of approximately 3.50 mg/mL, which is comparable to the IC_50_ of the hexapeptide SVGGAL, extracted from skipjack tuna, which is approximately 5.59 mg/mL. Both peptides can interact with a range of amino acids near the active pocket of XOD. The N-terminal tryptophan (Trp) residue of ALSGSW may confer an advantage owing to its hydrophobic characteristics, which facilitate its embedding into the hydrophobic pocket of molybdopterin. Furthermore, fish meat protein has a higher utilization rate than other fish byproducts. The complex pretreatment process of fish byproducts may limit their potential applications.

Besides these marine animal-derived FBPs, some protein hydrolysate products, the peptide sequences of which remain to be identified, have XOD inhibitory activity owing to their complex composition, which may include oligopeptides, peptides, and free amino acids. For example, the hydrolysates of salamander bone powder [64] and Antarctic Krill meat [65] have been demonstrated to possess XOD inhibitory activity. The imitation sea cucumber is an invertebrate that is a rich source of high-quality protein, relatively low in fat, and abundant in various essential amino acids required by the human body. Despite the observation by Huang et al. that the dipeptides present in imitation sea cucumber exhibited a less pronounced XOD enzyme activity inhibition effect than tilapia, Sun et al. [66] observed that the protein hydrolysate of imitation sea cucumber upregulated the fructose 1, 6-bisphosphate aldolase gene in hyperuricemic rats, enhanced glycogenesis, and reduced the generation of uric acid precursors. This discrepancy may be attributed to the disparate targets and underlying mechanisms of FBP in in vitro enzymatic reaction systems and within animal disease models. Moreover, some peptides may undergo significant structural modifications following multiple degradations (such as those caused by pepsin and trypsin) within the body. This could cause significantly different outcomes from those observed in vitro. For example, the peptide YLDNY, identified from the alkaline protease digestion of shark cartilage water extract, did not demonstrate in vitro XOD inhibitory activity. However, it effectively reduced serum uric acid concentration in a hyperuricemic animal model. Further research has identified a peptide structure that yields three short peptides, DN, LDN, and DNY, which have been confirmed to have XOD inhibitory activity in vitro [67]. Nevertheless, it remains to be seen whether these three short peptides play a significant role in reducing uric acid levels in animals.

Marine plants, such as large algae, have evolved intricate and distinctive metabolic pathways to facilitate their adaptation to dynamic and variable marine ecosystems. Nevertheless, research on marine plants as a peptide source remains scarce. Bao [68] evaluated the XOD inhibitory effects of two algae water extracts and observed notable inhibitory activity, with IC_50_ values of 0.58 and 0.32 mg/mL, respectively. The water-phase fractions of these two algae contain a variety of components, including sugars, glycosides, peptides, proteins, and alkaloids. Despite evidence from previous studies indicating that certain algal polyphenols and polysaccharides can significantly inhibit XOD activity and exert hypouricemic effects, the potential of algal peptides to inhibit XOD and lower uric acid levels remains largely unexamined. Peptides derived from algal proteins, particularly those containing hydrophobic and aromatic amino acids, exhibit a range of biological activities [69]. Further investigation of algae as a protein source is warranted to facilitate the development of antihyperuricemic functional food products.

**Table 1:** Preparation methods and FBP sequences of marine animal protease hydrolysates with XOD inhibitory activity have been reported in the literature.

Classify	Source	Pretreatment of Raw Materials	Preparation Method	Peptide Sequence	XOD Inhibitory	References
Marine fishes	*Katsuwonus pelamis*	Fish pieces	55 °C, pH = 7.0, and hydrolyzed using 0.25% Alcalase 2.4 L (2.06 × 10^5^ U/mL)	FH	IC_50_: 25.7 mM	[70]
	*Skipjack Tuna*	The back-belly meat is made into minced meat	Neutroprotease hydrolysis	ACECD	IC_50_: 7.23 mg/mL	[71]
	*Auxis thazard*	The fish is made into minced meat	Autolysis conditions: pH = 7–9, 50–70 °C, 4–6 h: the ratio of 1:3, pH 8.1, 60.2 °C, add enzyme amount of 500 U/g, using alkaline protease hydrolysis for 5 h	PDL	IC_50_: 4.37 mg/mL	[72]
				SVGGAL	IC_50_: 5.59 mg/mL	
	*Sadinops sagax*	The fish is made into minced meat	The autolysis of fish protein: pH = 8.55 °C, 5 h; foreign enzyme conditions: liquid ratio was 1:2, pH = 9.60 °C, plus enzyme was 0.15%, alkaline protease for 4 h, and the supernatant was separated by 20 nm ceramic membrane	FLR	IC_50_: 0.499 mg/mL	[73]
				RPK	IC_50_: 0.712 mg/mL	
	Tilapia Skin	Base-treated fish skin sterile water was incubated with fish skin in the freeze-dried supernatant	pH = 80, 48 °C, 3.2% for 6 h	TSPW	IC_50_: 3.51 mg/mL	[74]
	*Scomberomorus niphonius*	Heat the Fish pieces to de-nature the enzyme	Stock ratio 1:3, 55 °C, 5% more enzyme, use papain hydrolysis for 6 h, remove the supernatant, centrifuge the middle layer, and adjust the pH to 7.0–7.4	IIAPPER	IC_50_: 6.08 mg/mL	[75]
				AGFAGDDAPR	IC_50_: 6.15 mg/mL	
	*Larimichthys polyactis*	The fish is boiled to denature the enzyme	papain hydrolysis	WDDMEKIW	IC_50_: 3.16 mM	[76]
				APPERKYSVW	IC_50_: 5.86 mM	
	*Scophthalmus maximus*	Heat the back meat pieces to denature the enzyme	Stock ratio 1:3, enzyme concentration 4.0%; trypsin: alkaline protease = 1:1	FSLVHYAGTV	IC_50_: 3.741 mg/mL	[77]
				FTNEKLQQFF	IC_50_: 4.406 mg/mL	
				WDDMEKIWHH	IC_50_: 2.203 mg/mL	
	Bass Myosin	Amino acid sequence of Myosin-7B protein (1936 amino acids) obtained from NCBI database	Virtual enzymatic digestion by PepideCultter with Trypsin (pH = 1.3)	DEEIDN	IC_50_: 0.503 mg/mL	[78]
	Tuna	Bone myosin and metamyosin sequences were obtained from the NCBI database	Pepsin, trypsin, and chymotrypsin were selected to cleave the peptide on the ExPASy PeptideCutter program order	EEAK	IC_50_: 173 ± 0.06 μM	[79]
				FH	IC_50_: 257.0 μM	
	Bonito	Chyrime was assessed by heating (Bonito surimi protein was separately mixed with distilled water and preheated at 100 °C for 5 min to inactivate the enzymes)	Materials were mixed with distilled water at a ratio of 10% (*w*/*w*), pH = 7.0, 55 °C, 0.35% added enzyme, and hydrolyzed using papain for 4 h	WML	The inhibitory activity at 20 mM was 40%	[80]
	*Oreochromis mossambicus*	The flesh of fish	The liquid ratio was 1:9,56 °C, 3% enzyme, and hydrolyzed by trypsin and alkaline protease at 1:2 for 4 h	KE	The inhibitory activity at 40 mg/mL was 49.0%	[63]
	*Decapterus maruadsi*	Fish pieces	The liquid ratio was 1:2, pH = 7.0, 0.3%, 50 °C, and neuproteinase hydrolysis for 6 h	KGFP	The inhibitory activity at 5 mM was 5.43%	[81]
				FPSV	The inhibitory activity at 5 mM was 22.61%	
				FPFP	The inhibitory activity was 20.09% at 5 mM,	
				WPDGR	The inhibitory activity at 5 mM was 16.21%	
	*Trachinotus ovatus*	The flesh of fish	The liquid ratio was 1:3, pH = 7.0, 54 °C, plus enzyme was 0.19%, and neutral protease was used for 3.85 h	FPAW	IC_50_: 3.81 ± 0.18 mM	[82]
				LLPW	IC_50_: 4.17 ± 0.12 mM	
				WLLP	IC_50_: 43.06 ± 0.73 mM	
				FHLP	IC_50_: >50 mM	
	*Thunnus orientalis*	The myosin sequence was obtained from the NCBI (GenBank: BAL27686.1)	Myosin amino acid sequence release peptide sequence was cut using pepsin and trypsin in the ExPASy program	ICRK	IC_50_: 14.18 mg/mL	[83]
				FDAK	IC_50_: 16.8 mg/mL	
				MMER	IC_50_: 15.3 mg/mL	
	*Gadus macrocephalus Tilesius*	The bone and bone mixture were dried and ground to powder	The liquid ratio is 1:5, the enzyme amount is 600 U/g, 45 °C, without changing the system pH conditions, and the alkaline protease hydrolysis for 8 h	FF	IC_50_: 0.80 mM	[84]
				YF	IC_50_: 0.52 mM	
				WPW	IC_50_: 1.68 mM	
				WPDAR	IC_50_: 0.40 mM	
				YNVYGW	IC_50_: 0.23 mM	
	*Prionace glauca*	After hydration, the dried fin cartilage was peeled, dried, and the abrasive liquid ratio was 1:5. The suspension was collected twice, and the supernatant was extracted with cold water and combined with the supernatant twice	Solution ratio 1:12.5, pH = 8.0, 0.04%, 60 °C, using alkaline protease reaction for 4 h	DN	500.0 μM	[67]
				LDN	760.0 μM	
				DNY	880.0 μM	
				LDNY	750 μM	
Invertebrate	*Ostrea rivularis Goul*	Stir the oyster meat into a broken meat puree	The ratio of 1:5, pH = 2.0, 40 °C, and 3000 U/g were hydrolysis using acid protease for 4 h	GGYGIF	IC_50_: 4.28 mM	[85]
				ALSGSW	IC_50_: 2.17 mM	
				MAIGLW	IC_50_: 3.48 mM	
				GGWGIG	IC_50_: 2.37 mM	
				WGYGIF	IC_50_: 2.21 mM	
				WGGGW	IC_50_: 2.07 mM	
				WGWGIG	IC_50_: 1.96 mM	
				WGWGW	IC_50_: 1.86 mM	
	Pacific White Shrimp	The database of complete protein sequence was constructed by searching	1330 oligopeptides were selected from the database for docking with XOD, and 653 potential XOD inhibitory peptides were obtained by virtual screening	YNITGW	IC_50_: 9.78 mM	[86]
				GDEY	20.67 mM	
				AGDY	21.82 mM	
				PDARG	47.35 mM	
				WYNITGWW	23.99 mM	
	*Apostichopus japonicus*			GPAGPR	The inhibitory activity at 40 mg/mL was 37.3%	[87]
				GPSGRP	The inhibitory activity at 40 mg/mL was 46.8%	

## 6. Methods for Obtaining Antihyperuricemic Peptides from Marine Organisms

The specific sequence and structure of bioactive peptides determine their diverse biological activities and activity levels [88]. Enzymatic hydrolysis can yield peptides with specific therapeutic effects. During this process, polypeptide chains within protein structures are cleaved, resulting in hydrolysis products with enhanced physicochemical properties, biological functions, and sensory characteristics [89]. The number of effective amino acid fragments in the proteolytic products is markedly increased, the preparation efficiency is enhanced, and the cleavage specificity can be optimized by fine-tuning the methods.

Bioinformatics represents a high-throughput screening method for targeting active peptides, facilitating the acceleration of the screening process for bioactive peptides and providing a novel research platform for elucidating the interaction mechanisms between proteins and peptides. To prepare hypouricemic active peptides, advanced technologies such as virtual screening, molecular docking, and molecular dynamics simulation can be used to predict, verify, and analyze the activity and efficacy of peptides, providing a reliable data foundation for elucidating the structure–activity relationship of peptides and XOD. Furthermore, a comprehensive investigation of the interaction patterns between peptides and the active center of XOD is essential for the exploration and optimization of their activity and efficacy. The initial step was to discover and prepare peptides with natural hypouricemic activity. Further optimization of the peptide structure may be achieved through the introduction of modifications, including the addition, deletion, or substitution of amino acids, to enhance peptide binding affinity to XOD.

### 6.1. Preparation of Bioactive Peptides via Enzymatic Hydrolysis

The traditional method for protein hydrolysis is enzymatic. The principal advantage of enzymatic digestion is its capacity to maintain the activity of bioactive peptides and their nutritional components, while ensuring safety, simplicity of operation, and broad applicability. Before hydrolysis, the raw materials were subjected to pretreatment. This includes the removal of marine fish byproducts (fish skin, fish bones, and viscera) and the extension of the shelf life of raw materials through high-temperature treatment, cutting, grinding, air-drying, and freeze-drying. These preliminary treatments prolong the storage life of the raw materials and are conducive to complete hydrolysis. It is noteworthy that different raw materials have disparate pretreatment requirements, particularly for fish byproducts. Fish skin contains a variety of non-collagenous substances, including fats, unsaturated fatty acids, vitamins, and trace elements. Therefore, the extraction of fish skin collagen necessitates the removal of these non-collagenous substances and the degradation of the collagen itself [90]. It is imperative that the collagen extracted from fish bones is free of residual fat molecules and ash [91]. The primary factors to be considered when optimizing hydrolysis conditions are hydrolysis time, temperature, pH, enzyme type, enzyme addition, and material–liquid ratio. To optimize the hydrolysis scheme, experiments are often conducted using single-factor, response surface, or orthogonal experiments. The most important indicators are the hydrolysis degree sedimentation rate, peptide yield, peptide production, and XOD inhibition rate.

The hydrolysis products of proteins typically comprise peptides of varying lengths and free amino acids, which can be used directly to ascertain the XOD inhibitory activity and hypouricemic effects in animal models. However, these effects are frequently the consequence of the collective influence of numerous peptides, rendering the investigation of their underlying mechanisms and identification of specific action targets a formidable challenge. Moreover, protein hydrolysis products derived from marine animal sources may contain purines, which can elevate uric acid levels when inappropriate doses of drugs are administered to verify their effects in vivo [92]. Therefore, it is imperative to purify and identify specific bioactive peptides from the protein hydrolysis products.

Ultrafiltration, ion exchange chromatography, and gel filtration chromatography (GFC) are effective techniques for separating peptide mixtures with varying molecular weights. The combination of mass spectrometry technology with peptide separation techniques allows the visualization of peptide sequences, thus facilitating the identification of specific peptides within a mixture. For example, Zhao et al. [85] used ultrafiltration membranes with molecular weight cut-offs of 10 and 5 kDa to remove impurities and salts from oyster hydrolysate. Subsequently, the authors used GFC to successively elute and identify fraction X3, which exhibited the highest XOD inhibitory activity. This fraction was enriched with peptides that exhibited the highest XOD inhibitory activity. In another study, cation exchange chromatography was used to ascertain that neutral or weakly basic peptides exhibited greater XOD inhibitory activity than acidic peptides. Subsequently, a Superdex Peptide-10/200 GL gel filtration column was used to quantify the XOD inhibitory activity of each fraction (Frc1–Frc5). The inhibitory activity of Frc2 was higher than the original tuna hydrolysate mixture, substantiating the efficacy of this method for the separation and purification of protein hydrolysis products. Ultimately, four potential active peptide segments were identified using liquid chromatography–tandem mass spectrometry (LC-MS/MS): PGACSN, WML, AMPF, and FGVG [80].

The use of enzymatic hydrolysis as a safe and straightforward method can influence the molecular weight distribution and quality of the final product because of the selectivity and active sites of the proteases used. The selectivity of hydrolyzed peptide tendons allows for the classification of proteases as endopeptidases or exopeptidases. For example, the peptide sequences WML [81], ACECD [71], FH [70], PDL [72], and MMIMLEPL [93] were obtained through the specific cleavage of sea fish proteins by endopeptidases. The peptides displayed notable discrepancies in their XOD inhibitory and hypouricemic activities, which can be attributed to disparate peptide bond cleavage sites. Therefore, it is evident that hydrolysis is not a singular and repetitive process; even when the same protease and substrate are used, it is difficult to guarantee consistency in the peptide sequences present in the enzymatic hydrolysis products. To address the limitations of traditional hydrolysis methods, alternative techniques have been proposed, including ultrasonic-assisted hydrolysis [94], microwave-assisted acid hydrolysis [95], high hydrostatic pressure (HHP) [96], and subcritical water hydrolysis [97]. However, these methods are typically less effective than traditional methods and are best used as supplementary techniques along with enzymatic hydrolysis. Consequently, the development of methods to obtain FBPs using computer technology has become a common trend.

### 6.2. Obtaining Peptides by Combining Peptidomics and Bioinformatics

Several FBP databases have been constructed, including those for protein sequences, protein virtual hydrolysis servers, and bioactive peptides [60]. These databases have amassed tens of thousands of protein sequences, establishing a robust foundation for the development of peptidomics. Peptidomics, a subfield of proteomics, uses a “bottom-up” approach to directly identify peptide segments within specific samples. In 2008, the concept of food peptidomics was first proposed, defining an efficient identification technology involving the preparation and purification of bioactive peptides in food, the interaction mechanism between bioactive peptides and receptors, and the structure–activity relationship [98]. Peptidomics represents an emerging field with the potential to bridge the domains of proteomics and metabolomics. It can elucidate the intricate chemical structures of diverse peptides and provide insights into the synthesis and degradation of proteins under various physiological and pathological conditions. When identifying XOD inhibitory and hypouricemic peptides, peptidomics is frequently used as a robust novel peptide identification tool, integrating techniques such as mass spectrometry analysis and high-throughput sequencing with computer simulation technologies such as molecular docking and virtual screening.

Computer simulation technology allows for the rapid generation of new hypotheses, and has been extensively used in the prediction and design phases of novel peptide development. Virtual screening uses computer simulation to predict interactions between peptides and targets based on various parameters, including the strength of affinity, number of hydrogen bonds, number of key amino acids, and their positions in the peptide sequence. This allows for the evaluation and selection of potential compounds. Virtual screening can be classified according to the interaction mode between receptors and ligands and can be divided into receptor- and ligand-based virtual screening. Molecular docking is the most frequently used virtual screening method. Mao et al. [86] initiated the process by utilizing the three-dimensional structure of the target protein (XOD) to predict the ligand binding sites on the receptor surface through molecular docking. The authors demonstrated that peptides exhibiting heightened XOD inhibitory activity usually have aromatic or hygrophobic amino acids at their extremities. A reverse screening method based on surface plasmon resonance (SPR) ligand fishing technology can identify potential peptides. This method involves immobilizing XOD particles and fishing ligands that bind to XOD in hydrolysate samples [75,77]. Another ligand-based virtual screening method, quantitative structure–activity relationship (QSAR), is frequently used in the virtual screening of functional biomolecules [60]. This entails establishing a statistical correlation between chemical structure descriptors, properties, and biological activity of molecules based on a set of known bioactive peptides [99,100]. Despite the absence of a mature QSAR model for screening antihyperuricemic FBPs, the QSAR method has yielded successful practical results in the prediction of new XOI and URAT1 inhibitors [101,102].

Optimization of peptide chain structures can be achieved through the replacement of amino acids, a process that exemplifies the application of bioinformatic technology in drug design. The active site of XOD comprises 18 amino acids, among which Glu802, Arg880, Phe914, Phe1009, and Glu1261 have been shown to block the active site of enzyme catalytic inhibitors [101]. By modifying the peptide chain length and increasing the proportion of key amino acids, the effective interactions between peptides (such as the number of hydrogen bonds and π-π stacking) can be enhanced, increasing the biological activity of peptides [67,85,103]. The integration of peptidomics and bioinformatics has enhanced the efficacy of the preliminary screening and identification of active peptides. However, certain constraints persist. For instance, the outcomes of computational simulations merely disrupt the primary structure of proteins, whereas the more intricate effects of protein secondary and tertiary structures on biological activity remain unclear. Further experimental studies must substantiate the veracity of the computer-generated results [104]. Table 2 provides a list of URLs for protein information databases, peptide databases, computer simulation tools, and peptide property prediction tools.

## 7. Evaluation of the Antihyperuricemic Effects of Marine-Derived Bioactive Peptides

### 7.1. Assessment of XOD Inhibitory Activity of Marine-Derived Peptides

XOD inhibitory peptides obstruct the active site of XOD with substrates through competitive or non-competitive means, inhibiting uric acid production. Given that uric acid exhibits a distinctive absorption peak within the 290–295 nm range (i.e., the maximum absorption wavelength), a reaction system comprising xanthine oxidase and hypoxanthine can be established. This system allows for the assessment of the biological activity of XOD inhibitory peptides through the measurement of uric acid absorbance or generation rate. The method of characterizing peptide properties by measuring absorbance is the UV method, which is a commonly used technique for characterizing peptide properties. However, hypoxanthine, as a substrate, exhibits a maximum absorption wavelength in the range of 267–272 nm. Therefore, unreacted hypoxanthine present in the system may interfere with the absorbance at 290–295 nm. The components of protein hydrolysates are complex, and besides peptides, they may contain polyphenols and flavonoid substances, which may have a significant UV absorption at 290–295 nm. The formula for the UV method is as follows:

XOD inhibitory percentage=1−Δx/Δy×100%

where Δ*x* is the net absorbance of uric acid in the system in the absence of inhibitors and Δ*y* is the net absorbance of uric acid in the system after the addition of inhibitors.

The second method is high-performance liquid chromatography (HPLC), which enables the quantitative analysis of uric acid content in a mixture following the completion of enzymatic reactions. This method offers a robust alternative to the UV method, particularly for compounds that exhibit spectral interference in the UV region when evaluating protein extracts containing polyphenols and active peptides containing tryptophan [70,105]. The formula for HPLC is as follows:

XOD inhibitory percentage=(A−B)/A×100%

where *A* refers to the uric acid content in the system in the absence of inhibitors and *B* refers to the uric acid content in the system after the addition of inhibitors.

### 7.2. Evaluation of the Hypouricemic Effect of Marine-Derived Peptides

The preliminary screening step for determining XOD activity inhibition by FBP may provide insight into the potential hypouricemic effects observed at the cellular level, with peptides exhibiting stronger XOD inhibitory effects showing more significant outcomes [106].

Notably, the mechanism of action of peptides in the human body is not limited to targeting a single XOD enzyme. Cell models can be used to simulate the local in vivo environment, facilitating the assessment of peptide efficacy, including safety, hypouricemic efficacy, membrane permeability, and digestive stability. To a certain extent, animal models of hyperuricemia can be used to simulate the complexity of human hyperuricemia. The selection of animals with uric acid metabolism mechanisms analogous to those observed in humans can enhance the similarity and reliability of research. The construction of hyperuricemia models typically uses a range of methodologies, including promotion of uric acid synthesis, inhibition of uric acid excretion, inhibition of uricase activity, and genetic modification of relevant biological systems.

#### 7.2.1. Hyperuricemia Cell Models

The liver is the primary site of uric acid synthesis, whereas the kidney is primarily responsible for uric acid excretion, with a minor portion being metabolized by the intestine. Consequently, the liver, kidney, and intestinal cells are optimal candidates for the construction of hyperuricemia cell models. Bisphenol A activates XOD in primary mouse liver cells, promoting uric acid synthesis. The results of molecular docking indicated that Asp360 and Lys422 on XOD were the preferred docking sites for BPA on the surface of XOD. Mutations at these two sites can block the actions of BPA and XOD [107]. In rat liver cells (BRL-3A), the use of 4 mM hypoxanthine as an inducer has been demonstrated to stimulate XOD activity, increasing the concentration of uric acid in the cell supernatant and promoting the development of a hyperuricemia model [108]. However, there are genetic differences in expression between mouse- and human-derived cells. Consequently, normal human liver cells (L02 cells) are widely used. Supplementation of L02 cell culture medium with 0.8 mM inosine or adenosine (2 mM) increased the concentration of uric acid precursors in cells. Furthermore, the successful construction of a hyperuricemia model has been achieved through the addition of exogenous XOD [109,110]. Following inosine treatment of L02 cells for 24 h, there was a notable increase in HGPRT mRNA levels, which elevated the guanine content involved in uric acid synthesis [111]. The uric acid synthesis pathway depends on the action of multiple enzymes (Figure 1). The L02 hyperuricemia cell model is an invaluable tool for elucidating the underlying mechanisms of excess uric acid synthesis in liver cells, which is crucial for the identification of novel drug targets.

The kidney plays a pivotal role in uric acid metabolism, with uric acid reabsorption and excretion primarily regulated by this organ. HK-2 cells (human renal tubular epithelial cells) retain the functional characteristics of the proximal tubule epithelium, which is a healthy kidney cell type. The addition of adenosine, adenine, and hypoxanthine as inducers to the HK-2 cell line under the catalysis of ADA and PNP enzymes results in the generation of uric acid precursors. Hou et al. optimized the modeling conditions of the HK-2 hyperuricemia cell model and determined that HK-2 cells inoculated at a density of 105 cells/mL for 24 h and in 2.5 mM adenosine for 30 h, and then incubated with a final concentration of 0.005 U/mL xanthine oxidase for 8 h, could generate a cell model with high adenosine utilization, a large amount of uric acid precursor generation, and complete enzymatic reaction [112]. The kidneys contain a variety of important uric acid transport proteins (Figure 2). The HK-2 hyperuricemia cell model can reflect the regulatory effect of peptides on intracellular uric acid levels and facilitate exploration of the potential mechanisms of peptides in the cell damage repair process. The peptide ADBP, derived from the bones of the large salamander, regulates the levels of urate salt reabsorption proteins (URAT1 and GLUT9) and increases the level of urate salt excretion protein (ABCG2) in HK-2 hyperuricemia cells, maintaining equilibrium in cellular uric acid metabolism. In addition, ADBP can facilitate the restoration of the cellular redox balance by repairing oxidative damage, promoting the repair of cellular damage [64].

The consumption of fructose has been demonstrated to induce hyperuricemia in HK-2 cells. This phenomenon occurs due to ketohexokinase-mediated fructose metabolism, which increases adenine deaminase activity and subsequently enhances uric acid production. Moreover, fructose may contribute to the development of inflammation by stimulating the expression of related inflammatory proteins and molecules, which reduce uric acid excretion and cause cell damage [113,114]. The hallmark of cellular damage is the cessation of cell proliferation, alterations in cell morphology, apoptosis, and shedding of cells. The utilization of cell models offers a straightforward, expeditious, and highly reproducible experimental platform for screening antihyperuricemic bioactive peptides. Two-dimensional disease models cannot fully replicate the intricacies of human physiological and pathological states. The construction of animal disease models has addressed this deficiency.

#### 7.2.2. Hyperuricemia Animal Models

Animal models of hyperuricemia serve as invaluable tools for the development of novel peptides. Rodents, particularly rats and mice, are frequently used as model carriers. Methods for constructing hyperuricemia animal models can be classified into two categories: genetic engineering modification and chemical drug induction. The uricase enzyme is present in rats and mice, resulting in significantly lower blood uric acid levels than those in humans. The gene encoding uricase can be knocked out to silence uricase expression, allowing animals to spontaneously develop hyperuricemia. The researchers used transcription activator-like effector nuclease technology to inactivate the uricase gene in mice with a pure C57BL/6J background, establishing a stable hyperuricemia mouse strain. This strain exhibits a low mortality rate and can maintain a stable increase in uric acid levels for 62 weeks [115,116]. A liver-specific conditional knockout Uox-defective mouse model was developed using the Cre/loxP gene targeting system. These mice exhibit uric acid levels comparable to those observed in humans and a normal lifespan, rendering them an effective model for evaluating hyperuricemia [117]. Moreover, the creation of hyperuricemia models is possible through the knockout of genes encoding uric acid transport proteins [118,119]. Notably, mice exhibiting hyperuricemia accompanied by early onset nephropathy, which is like acute hyperuricemia nephropathy in humans, have been produced by complete knockout of the *SLC2A9* gene, which encodes GLUT9 [118].

Direct supplementation with uric acid, adenine, or uric acid precursors has been shown to elevate serum uric acid levels in mice, resulting in the development of hyperuricemia. Nevertheless, the presence of uricase in animals renders this method unsuitable for constructing long-term stable hyperuricemia models [120,121,122,123]. The intake of yeast, fructose, or fat, either alone or in combination with several drugs, has been demonstrated to increase serum uric acid levels in mice and rats. These models can simulate complex disease models in which hyperuricemia coexists with one or more comorbidities; however, they are not suitable for studying pure hyperuricemia [124,125,126,127].

Oxonic acid potassium has been demonstrated to competitively inhibit uricase activity and increase uric acid levels. Administration over an extended period can cause the establishment of a sustained and stable hyperuricemia model. Nevertheless, oxonic acid potassium can only inhibit a portion of uricase activity, and the efficacy of increasing uric acid levels is constrained. For instance, the administration of 2% oxonic acid potassium to rats over a seven-week period has been demonstrated to induce only mild hyperuricemia [128]. The combined modeling method is an effective approach for rapidly increasing serum uric acid levels, extending the duration of disease, and reducing kidney damage, making it a commonly used method in research. For instance, a combination of yeast extract and 750 mg/kg oxonic acid potassium was administered to rats for 35 days, resulting in a serum uric acid level four times higher than the control group [129].

Hypoxanthine (250 mg/kg) and oxonic acid potassium (250 mg/kg) were administered intragastrically to the mice for 2 weeks. The serum uric acid level of the mice was 1.9 times higher than the control group and the liver uric acid level was 5.3 times higher [130].

Zebrafish exhibit physiological and biochemical characteristics analogous to those observed in mammals, and their disease-related signal transduction pathways are highly conserved in humans. Furthermore, zebrafish are relatively small and easy to operate and handle, which has contributed to their growing popularity as a high-throughput screening model [131,132,133]. The liver of zebrafish is fully developed and functional approximately five days after fertilization [134]. A combination of oxonic acid potassium (200/300/400 μM) and xanthine sodium salt (10/15/20 μM) has been used to construct an acute hyperuricemia model in 5-day-post-fertilization zebrafish larvae [135].

The mechanisms of action of peptides in animal models are complex and diverse, typically involving the inhibition of various enzymes. Antarctic krill peptides have been demonstrated to significantly reduce uric acid levels in mice by inhibiting XOD and ADA activity and by regulating urate salt transporter transcription to promote uric acid excretion by the kidney and intestine. The hypouricemic bioactive peptide ATO derived from skipjack tuna was used by researchers to intervene in hyperuricemic mice, resulting in the inhibition of XOD and ADA enzyme activity and a notable reduction in serum uric acid and blood creatinine levels [72]. The hypouricemic active peptide TP, isolated from the collagen of tilapia skin, has been demonstrated to significantly reduce ADA and XOD activity in the liver of mice. Furthermore, there was no significant difference in this reduction compared to the normal group, which indicates that TP effectively inhibits the transformation of purine substances into uric acid. In addition, TP has been demonstrated to reduce the activity of glutamic-oxaloacetic transaminase and glutamic-pyruvic transaminase, indicating a potential protective effect against acute liver injury [74]. Table 3 shows the effects of protein hydrolysates and FBPs derived from select marine animals on hyperuricemia animal models in vivo. The activities of liver XOD and ADA, as well as the expression of urate salt transport proteins, elucidate the mechanisms underlying uric acid homeostasis and regulation of the uric acid transport system, respectively. The expression level of inflammatory factors serves as a marker for evaluating the anti-inflammatory effects of protein hydrolysates or FBPs on the renal and hepatic inflammatory responses.

**Table 3:** Animal models, doses, and results of marine antihyperuricemic FBPs. ↑: Elevated levels or upward adjustments. ↓: Decrease in level or downward adjustment.

Source	Medicinal	Model and Molding Mode	Peptide Dose	Changes in Gene Expression	References
*Oreochromis mossambicus*	KE peptide	Male C57BL/6 mice; 10% fructose in drinking water for 12 weeks	10 mg/kg/day (GAspp)	secrum UA, BUN, and Cr ↓, ADA activities and XOD activities ↓; renal protein expression of GLUT9 and URAT1 ↓, ACBG2 ↑; renal mRNA level of ULUT9 and URAT1 ↓, mRNA level of ABCG2 ↑; renal inflammatory biomarkers (IL-1β and TNF-α ↓, IL-10 ↑); renal mRNA level of IL-1β, ASC, caspase-1 and NLRP3 ↓; renal protein expression of MyD88, ASC and caspase-1 ↓	[63]
*Katsuwonus pelamis*	Proteolysis product TPH mixed peptide	Adult SD rat; rat 2 g (DW)/kg (BW)) with potassium gavage for 1 week	83 mg DW/kg BW (gavage)	scrum UA ↓, XOD activities ↓	[70]
*Auxis thazard*	Proteolytic product ATO mixed peptides with molecular weight < 1 kDa	Male KM mice; 25 mg/mL potassium gavage for 1 week	600/300/150 mg/kg/day (by gavage)	secrum UA, Cr, and BUN ↓; hepatic XOD activities and ADA activities and MDA activities ↓, SOD activities and CAT activities ↑; renal mRNA level of GLUT9 and URAT1 ↓, mRNA level of ABCG2 and OAT1 ↑; inflammatory biomarkers (IL-1β MCP-1 and TNF-α) ↓	[72]
*Sadinops sagax*	Proteolytic product SSP mixed peptide	KM male mice; 250 mg/kg potassium and 5 g/kg yeast combined gavage for 3 weeks	600/300/150 mg/kg/day (by gavage)	secrum UA, Cr, and BUN ↓, hepatic XOD activities ↓; renal mRNA level of GLUT9 and URAT1 ↓, mRNA level of ABCG2 ↑; inflammatory biomarkers (Casp-3, IL-1β and NLRP3) ↓	[73]
Tilapia Skin	TSPW peptide	KM mice, 75 mg/kg/day adenine by gavage for 4 weeks	450/150/50 mg/kg/day (by gavage)	secrum UA, Cr, and BUN ↓; liver function indicators (GOT and GPT) ↓; hepatic XOD activities, ADA activities, and MDA activities ↓, SOD activities and CAT activities ↑; renal protein expression of URAT1↓	[74]
Bonito	Proteolysis product BH mixed peptide	SD rats; 2 g/kg (BW) potassium gavage for 1 week	300 mg/kg (by gavage)	secrum UA ↓, XOD activities↓	[80]
Tuna	Proteolytic product TMOP oligolytides	Male ICR mice; 200 mg/kg/day hypoxanthine and 30 mg/kg/day yeast extract by gavage for 8 weeks	300/500 mg/kg/day (by gavage)	secrum UA, BUN, and Cr ↓, XOD activities ↓; hepatic ADA activities, PNP activities, and PRPP activities ↓; renal mRNA level of GLUT9, URAT1, TNF-α, IL-1β and IL-6 ↓, renal mRNA level of ABCG2, SOD, GSH-Px and CAT ↑; inhibited the activation of NLRP3 inflammasome and TLR4/MyD88/NF-κB/ signaling pathways; suppressed the phosphorylation of p65-NF-κB	[136]

## 8. Structure–Activity Relationship of XOD Inhibitory Activity and Antihyperuricemic Peptides

Amino acids constitute the fundamental units of peptide chains and the structural attributes of marine-derived FBPs directly influence their XOD inhibitory activity. Low-molecular-weight peptides, particularly dipeptides, serve as pivotal molecular units that elicit XOD inhibitory effects [137]. Recent studies have demonstrated the potential of basic amino acids and basic dipeptides/tripeptides to reduce the formation of purines, pyrimidines, and uric acid crystals. Of these, tryptophan (Trp)-containing dipeptides have been identified as promising XOD inhibitors [137,138]. On the one hand, dipeptides, and tripeptides can resist the action of certain enzymes within the body, maintaining their complete physiological activity and morphology when absorbed into the bloodstream. Conversely, tryptophan (Trp), which acts as a non-competitive inhibitor of XOD, is a pivotal amino acid in peptide–protein interactions. The bioactivity of peptides is contingent on their chemical structure, size, and amino acid composition. The use of different proteases in the hydrolysis of the same raw material results in the production of hydrolysates with varying XOD inhibitory activity [72]. Accordingly, protease selection directly affects the quantity and quality of the peptides produced.

Alkaline protease is a commonly used serine endopeptidase that cleaves the carboxyl group of hydrophobic aromatic amino acids, resulting in the production of peptide segments with hydrophobic characteristics and smaller peptides [139,140]. The hydrolysis of tuna with alkaline protease yielded dipeptides FW and FH with a cyclic structure of amino acids at the N-terminus, which exhibited superior XOD inhibitory activity compared with 11 other peptides [70]. This finding is consistent with the results of another study that indicated a higher probability of XOD inhibitory activity when aromatic amino acid residues were located at the N-terminus of dipeptides/tripeptides [141]. Molecular docking results demonstrated that hydrophobic amino acids are more likely to enter the hydrophobic cavity near the active center of XOD, altering its spatial structure and forming a stable complex with XOD. This process prevents other purines from entering the channel and binding to the enzyme, preventing the production of uric acid [70,80].

The amino acid composition of marine organisms provides a rich source of potential XOI candidates for discovery from protein hydrolysates [64,142]. Under suitable conditions, acid proteases preferentially cleave hydrophobic and aromatic amino acids. Zhao et al. [85] used acid protease to facilitate the hydrolysis of oysters and identified three peptides with XOD inhibitory activity, namely GGYGIF, ALSGSW, and MAIGLW. All three peptides possess a C-terminal Phe or Trp residue. In addition, the authors indicated that peptides comprising alternating aromatic amino acids and some structurally simple amino acids connected in a moderate-length sequence demonstrated promising in vitro XOD inhibitory activity. The specificity of pepsin, which preferentially cleaves hydrophobic amino acids such as tryptophan, tyrosine, and phenylalanine at the N-terminus of proteins, is influenced by the amino acids at protein cleavage sites [143,144]. Assessment of the antigastrointestinal digestion capabilities of anionic peptides often uses gastric and pancreatic proteases, which are crucial digestive enzymes. It is postulated that peptides that survive the simulated “stomach-intestinal” system digestion are more accurately indicative of their antihyperuricemic activity.

## 9. Role of Antihyperuricemic Peptides in Alleviating Damage

In certain pathological conditions, such as tissue ischemia, hypoxic stress stimulates the production of elevated levels of xanthine oxidase and reactive oxygen species (ROS). During ischemia–reperfusion, molecular oxygen is transferred back to xanthine molecules, which then react with hypoxanthine and xanthine to produce substantial quantities of superoxide anions and hydrogen peroxide, exacerbating endothelial cell damage [145]. The production of free radicals may be inhibited by small-molecular-weight peptides by binding with Fe^2+^, enhancing the DPPH radical scavenging ability of protein hydrolysates [146]. Aromatic amino acids, histidine (His), and glycine (Gly) in small-molecular-weight peptides may play a significant role in this process [147,148,149].

In addition, uric acid has been demonstrated to function as an activator, inducing inflammatory responses through specific signaling pathways, which can cause damage to organs, such as the kidneys and liver [150,151,152]. It has been demonstrated that the hypouricemic effects of peptides can not only facilitate salutary alterations in biochemical markers associated with uric acid metabolism (XOD, ADA, uric acid, and creatinine), but also regulate the levels of pivotal elements in signaling pathways, attenuating inflammation. The NLRP3 inflammasome is a sensor of cellular stress that, along with the adaptor protein ASC and the effector protein caspase-1, constitutes a large multiprotein complex [153]. In the absence of external stimuli, the NLRP3 inflammasome is in an inactive state, complexed with the heat shock proteins HSP90 and SGT1. However, uric acid crystals have been observed to cause lysosomal damage and rupture, resulting in the release of tissue proteins and ROS, which induces inflammasome assembly and activation. KE and CE peptides, which were identified from the hydrolysates of tilapia, were observed to significantly suppress the transcription of NLRP3 and ASC in a mouse model that had been induced by fructose, reducing the level of inflammation [63].

**Figure 3:**
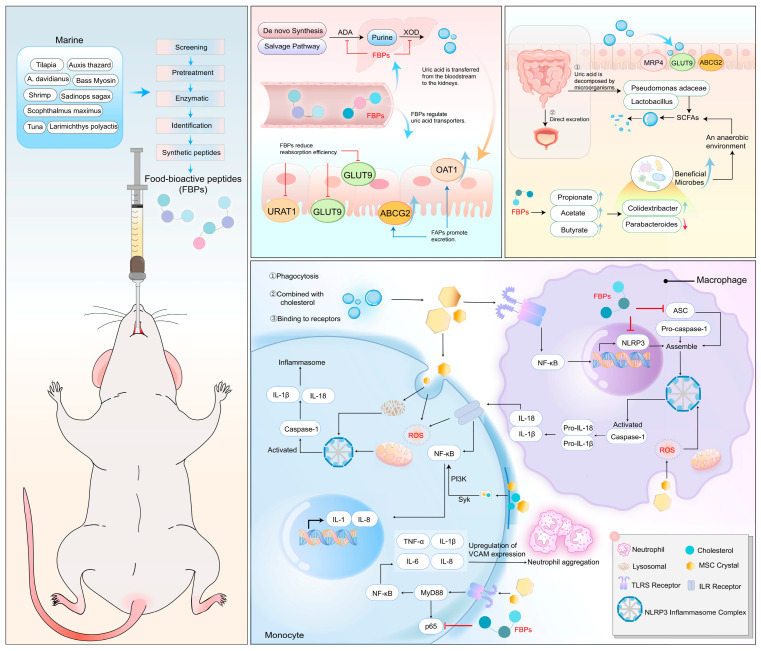
Schematic diagram of FBPs antihyperuricemia mechanism.

The TLR4 signaling pathway activates NF-κB, which produces pro-interleukin (IL)-1β, a primary substrate for caspase-1. In addition, KE and CE peptides have been observed to downregulate cytokine levels within the MyD88 signaling pathway, which is mediated by TLR4. This is achieved by inhibiting the phosphorylation of p65-NF-κB, which subsequently reduces the production of pro-IL-1β [63]. The administration of tuna meat oligopeptides (TMOP) effectively alleviated oxidative stress in the kidneys of hyperuricemic mice, accompanied by a notable reduction in the levels of representative inflammatory factors at the mRNA level. TMOP may also mitigate hyperuricemia and kidney inflammation by modulating the gut microbiota [136]. In Figure 3, we present a simplified illustration of the regulatory effects of antihyperuricemic peptides on uric acid within the synthesis and metabolic processes.

These findings indicate that marine animal protein hydrolysates and antihyperuricemic peptides can reduce the production of uric acid and ROS by inhibiting XOD enzyme activity or alleviating damage by regulating the expression of inflammatory factors in hyperuricemic animal or cell models. Nevertheless, research on the mechanisms of action of marine-derived FBPs in inflammation-related signaling pathways remains scarce and warrants further in-depth investigation to elucidate the underlying mechanisms.

## 10. Prospects for Developing Antihyperuricemic Bioactive Peptides

A variety of marine animals can be used as sources of FBPs with antihyperuricemic functions. This review focuses on the preparation and efficacy evaluation methods of antihyperuricemic marine-derived FBPs and analyzes the mechanisms of action of FBPs from the perspective of structure–activity relationships and improvement of pathological conditions in hyperuricemic animal models. To date, few studies have identified the principal components of protein hydrolysates derived from marine animals that affect hyperuricemic animal models. This lack of research has hampered the identification and clarification of specific effective components when discussing the hypouricemic mechanism. The presence of purines, polysaccharides, polyphenols, and other substances in hydrolysates may also result in erroneous conclusions.

Marine-derived FBPs offer nutritional and health benefits to organisms. Therefore, functional foods play a major role in the market transformation of FBPs. Although FBPs provide beneficial effects for the management of hyperuricemia from multiple perspectives, there is still scope for improvement to optimize their positive impact on humans. It is imperative that future studies determine how to ensure balanced nutrition and maintain the quality of life of patients while reducing blood uric acid levels. The low bioavailability of functional FBPs poses a significant challenge to their widespread utilization. In this study, we present several potential solutions.

Peptides ingested orally are initially digested by amylase and lipase in the saliva, followed by further breakdown in the stomach (by pepsin) and pancreas (by trypsin) into smaller dipeptides and tripeptides. These small-molecular-weight peptides appear to be more digestible than intact proteins and free amino acids [154], exhibit adequate membrane permeability, and can be absorbed by the intestinal wall and enter systemic circulation [155]. However, alterations in acidic and alkaline conditions of the gastrointestinal tract may cause peptide inactivation. Other peptide drugs that are available are typically administered intravenously to ensure that hypouricemic bioactive peptides can reach the intended target directly and in their original unaltered state. Most peptides exhibit high plasma stability and favorable clearance rates. Nevertheless, patient compliance remains a significant challenge, particularly in the context of chronic diseases necessitating prolonged treatment, such as hyperuricemia [58,156]. The available hypouricemic bioactive peptides are primarily short-chain dipeptides and tripeptides, which possess enhanced structural stability and can be administered orally. However, the combination of amino acids in short peptides is limited, and enhancing bioavailability by modifying only chain length is not a long-term solution. Peptides exhibiting robust XOD inhibitory activity possess a chain length between two and five amino acids. These peptides may use alternative methods to enhance bioavailability, such as microencapsulation, which has been demonstrated to preserve the high bioactivity of ACE peptides following gastrointestinal digestion [157]. This suggests that encapsulation technology may be an effective method for the protection and maintenance of the bioavailability of XOD inhibitory peptides. Microneedles (MNs) can penetrate the stratum corneum and form channels, facilitating direct drug delivery to the upper dermis or epidermis [158]. A previous study reported a soluble nanoneedle system encapsulating colchicine, which improved drug delivery efficiency and reduced drug loss through transdermal drug delivery [159]. Moreover, certain antihyperuricemic peptides have been shown to regulate the expression of inflammatory factors in inflammatory-related signaling pathways, exerting a local anti-inflammatory effect. Microneedles have the potential to become an effective local delivery method.

The establishment of hyperuricemia cell models is predominantly based on conventional two-dimensional cell techniques. The growth of cells in culture dishes or multi-well plates occurs in a single layer, facilitating observation of the status and changes in cell growth before and after drug treatment. However, the static nature of this culture method precludes its ability to fully replicate the complexity of real human organ tissues and cell-to-cell interactions, which are inherent limitations of 2D cell models. Consequently, the utilization of 2D cell models in the research and development of novel pharmaceutical agents frequently necessitates the use of animal models to assess drug toxicity and bioavailability. Three-dimensional cell models, established through suspension culture, permit cells to grow and migrate in a three-dimensional space. This in vitro culture technique compensates for the aforementioned deficiencies and should be applied in future research.

## 11. Conclusions

This review provides an overview of marine-derived bioactive peptides that have been shown to possess antihyperuricemic properties. In this review, we have explored the preparation methods, mechanisms of action, and potential applications of these peptides. Currently, the development of antihyperuricemic active substances is gradually expanding from marine fish to marine invertebrates, suggesting that a variety of novel bioactive peptide sequences with potential hypouricemic effects will be unveiled in the future. The application of computer technology offers new strategies for peptide structure design and optimization. However, the exploration of peptide–protein interactions is limited to the primary structure of proteins, indicating a need to further deepen the application of computer technology in this area. Concurrently, we have observed that the synthesis of these low-molecular-weight peptides is inefficient and costly. While optimizing the quality of peptide synthesis, increasing the production efficiency of peptides is also a matter that requires consideration. Peptides are characterized by high safety and a broad range of applicability, which gives them tremendous potential in the prevention and treatment of diseases. However, there have been no reports regarding the clinical trial phase of antihyperuricemic peptides. Future research in this area will probably enhance the current understanding and facilitate the development of more effective and safer therapeutic strategies for hyperuricemia.

## Figures and Tables

**Table 2:** Commonly used protein and peptide databases and computer simulation tools. The websites listed in the table are all professional sites and were accessible as of 30 November 2024.

Tool Type	Tool Name	Website
Protein Sequence Database	NCBI Protein	http://www.ncbi.nlm.nih.gov/protein/
	Uniprot	http://www.uniprot.org
	RCSB	http://www.rcsb.org/
	BioXFinder	https://bio.bcpmdata.com/home
	EMDB	http://www.ebi.ac.uk/emdb/
Bioactive Peptide Database	SATPdb	https://webs.iiitd.edu.in/raghava/satpdb/
	Pepbank	https://pubmed.ncbi.nlm.nih.gov/17678535/
	StraPep	http://isyslab.info/CBPS/index.php
	PlantPepDB	http://14.139.61.8/PlantPepDB/index.php
Protein Structure Database	BMRB	https://bmrb.io/
	SMR	https://swissmodel.expasy.org/repository/
	PDB	https://www.rcsb.org/
Protein Virtual Hydrolysis Server	Peptide Cutter	http://web.expasy.org/peptide_cutter/
	BIOPEP-UWM	https://biochemia.uwm.edu.pl/biopep-uwm/
	MAT	http://hazralab.iitr.ac.in/index.html
Peptide Virtual Screening Tool	BLAST	http://blast.ncbi.nlm.nih.gov
Peptide Virtual Screening, Molecular Docking, and Molecular Dynamics Simulation Tools	Discovery Studio visualizer 21.1.0.0	
	YASARA-Structure 21.8.27 version	
	Gromacs 4.5	
	Schrodinger 3.4	
Peptide Bioactivity Prediction Tool	PeptideRanker	http://distilldeep.ucd.ie/PeptideRanker/
Peptide Toxicity and Allergy Prediction Tool	ToxinPred	https://webs.iiitd.edu.in/raghava/toxinpred/index.html
Peptide Solubility Prediction Tool	Innovagen	http://www.innovagen.com/proteomics-tool
Peptide Physicochemical Property Prediction Tool	ProtParam	https://web.expasy.org/protparam/
Peptide Gastrointestinal Absorption Prediction Tool	SwissAMDE	http://www.swissadme.ch/index.php
Peptide Half-life Prediction Tool	Plifepred	https://webs.iiitd.edu.in/raghava/plifepred/batch.php

## References

[1] Rathinam R.B., Acharya A., Robina A.J., Banu H., Tripathi G. (2024). The immune system of marine invertebrates: Earliest adaptation of animals. Comp. Immunol. Rep..

[2] Ameen F., AINadhari S., AI-Homaidan A.A. (2021). Marine microorganisms as an untapped source of bioactive compounds. Saudi. J. Bio. lSci..

[3] Naylor R.L., Kishore A., Sumaila U.R., Issifu I., Hunter B.P., Belton B., Bush S.R., Cao L., Gelcich S., Gephart J.A. (2021). Blue food demand across geographic and temporal scales. Nat. Commun..

[4] Ismail B.P., Senaratne-Lenagala L., Stube A., Brackenridge A. (2020). Protein demand: Review of plant and animal proteins used in alternative protein product development and production. Anim. Front..

[5] Berrazaga I., Micard V., Gueugneau M., Walrand S. (2019). The Role of the Anabolic Properties of Plant- versus Animal-Based Protein Sources in Supporting Muscle Mass Maintenance: A Critical Review. Nutrients.

[6] Zang J., Xu Y., Xia W., Regenstein J.M. (2020). Quality, functionality, and microbiology of fermented fish: A review. Crit. Rev. Food. Sci. Nutr..

[7] Hamed I., Özogul F., Özogul Y., Regenstein J.M. (2015). Marine bioactive compounds and their health benefits: A review. Comp. Rev. Food Sci. Food Safe..

[8] Fang N., Lv L., Lv X., Xiang Y., Li B., Li C., He C., Chen W., Chen T., Zhou J. (2023). China multi-disciplinary expert consensus on diagnosis and treatment of hyperuricemia and related diseases, 2023 ed. Chin. J. Pract. Intern. Med..

[9] Zhu Q., Yu L., Li Y., Man Q., Jia S., Liu B., Zong W., Zhou Y., Zuo H., Zhang J. (2023). Association between dietary approaches to stop hypertension (DASH) diet and hyperuricemia among Chinese adults: Findings from a nationwide representative study. Nutr. J..

[10] Chu Y., Zhao Y. (2023). Research progress on the mechanism of action of sex hormones in hyperuricemia. Mod. Med. J..

[11] Yang X., Liu D., Zhao X., Han Y., Zhang X., Zhou Q., Lv Q. (2024). Hyperuricemia drives intestinal barrier dysfunction by regulating gut microbiota. Heliyon.

[12] Ren S., Li Q., Me C., Jiang D., Yu Y. (2023). Microbes participate in the synthesis, decomposition and transport of human uric acid. J. Microecol..

[13] Stamp L.K., Day R.O., Yun J. (2016). Allopurinol hypersensitivity: Investigating the cause and minimizing the risk. Nat. Rev. Rheumatol..

[14] Yaseen W., Auguste B., Zipursky J. (2023). Allopurinol hypersensitivity syndrome. Can. Med. Assoc. J..

[15] Mąsior M.N., Rostkowska O.M., Furmańczyk-Zawiska A.F., Wieczorek-Godlewska R., Wyzgał M., Durlik M. (2024). DRESS syndrome: Renal involvement in two cases—A comprehensive analysis and literature review of improved diagnosis and treatment. Am. J. Case Rep..

[16] Perdigão S., Alves A.S., Nunes M., Sousa C., Barros N. (2024). Allopurinol-induced toxic epidermal necrolysis. Cureus.

[17] Ma X., Shi M., Chen S., Zhong X., Lin C., Xu Q. (2024). Allopurinol is Associated with an Increased Risk of Cerebral Infarction: A Two-Sample Mendelian Randomization Study. ACS Omega.

[18] Miryam M.R., Carlos M.M., Frank S.L., Miguel M.G.J. (2023). Acute hepatitis secondary to febuxostat. Rev. Esp. Enferm. Dig..

[19] Ullah Z., Yue P., Mao G., Zhang M., Liu P., Wu X., Zhao T., Yang L. (2024). A comprehensive review on recent xanthine oxidase inhibitors of dietary based bioactive substances for the treatment of hyperuricemia and gout: Molecular mechanisms and perspective. Int. J. Biol. Macromol..

[20] Wang Y., Ma M., Huang Y., Fan S., Peng J., Li S., Su X., Wang Y., Lu C. (2024). Food-derived bio-functional peptides for the management of hyperuricemia and associated mechanism. Food Sci. Hum. Wellness.

[21] Cruz-Casas D.E., Aguilar C.N., Ascacio-Valdés J.A., Rodríguez-Herrera R., Chávez-González M.L., Flores-Gallegos A.C. (2021). Enzymatic hydrolysis and microbial fermentation: The most favorable biotechnological methods for the release of bioactive peptides. Food Chem. Mol. Sci..

[22] Zhang W., Boateng I.D., Xu J. (2024). Novel marine proteins as a global protein supply and human nutrition: Extraction, bioactivities, potential applications, safety assessment, and deodorization technologies. Trends Food Sci. Technol..

[23] Wang P., Zhang Y., An Y., Xu K., Xu X., Fu C., Lin J., Xu S., Li Q., Lei H. (2013). Protection of a New heptapeptide from Carapax trionycis against Carbon Tetrachloride-Induced Acute Liver Injury in Mice. Chem. Pharm. Bull..

[24] Chakrabarti S., Wu J. (2015). Milk-derived tripeptides IPP (Ile–Pro–Pro) and VPP (Val–Pro–Pro) promote adipocyte differentiation and inhibit inflammation in 3T3-F442A cells. PLoS ONE.

[25] Ding F., Qian B., Zhao X., Shen S., Deng Y., Wang D., Zhang F., Sui Z., Jing P. (2013). VPPIPP and IPPVPP: Two hexapeptides innovated to exert antihypertensive activity. PLoS ONE.

[26] Zhao W.H., Chi C.F., Zhao Y.Q., Wang B. (2018). Preparation, Physicochemical and Antioxidant Properties of Acid- and Pepsin-Soluble Collagens from the Swim Bladders of Miiuy Croaker (*Miichthys miiuy*). Mar. Drugs.

[27] Bank M.S., Duarte C.M., Sonne C. (2022). Intergovernmental panel on blue foods in support of sustainable development and nutritional security. Environ. Sci. Technol..

[28] Xiang Z., Xue Q., Gao P., Yu H., Wu M., Zhao Z., Li Y., Wang S., Zhang J., Dai L. (2023). Antioxidant peptides from edible aquatic animals: Preparation method, mechanism of action, and structure–activity relationships. Food Chem..

[29] So A., Thorens B. (2010). Uric acid transport and disease. J. Clin. Investig..

[30] Nyhan W.L. (2005). Disorders of purine and pyrimidine metabolism. Mol. Genet. Metab..

[31] Maiuolo J., Oppedisano F., Gratteri S., Muscoli C., Mollace V. (2016). Regulation of uric acid metabolism and excretion. Int. J. Cardiol..

[32] Lu Y., Zhang H., Han M., Wang P., Meng L. (2024). Impairment of Autophagy Mediates the Uric-Acid-Induced Phenotypic Transformation of Vascular Smooth Muscle Cells. Pharmacology.

[33] Lee T., Lu T., Chen C., Guo B., Hsu C. (2021). Hyperuricemia induces endothelial dysfunction and accelerates atherosclerosis by disturbing the asymmetric dimethylarginine/dimethylarginine dimethylaminotransferase 2 pathway. Redox Biol..

[34] Balakumar P., Alqahtani A., Khan N.A., Mahadevan N., Dhanaraj S.A. (2020). Mechanistic insights into hyperuricemia-associated renal abnormalities with special emphasis on epithelial-to-mesenchymal transition: Pathologic implications and putative pharmacologic targets. Pharmacol. Res..

[35] Kawasoe S., Kubozono T., Salim A.A., Ojima S., Yamaguchi S., Ikeda Y., Miyahara H., Tokushig K., Ohishi M. (2024). J-shaped association between serum uric acid levels and the prevalence of a reduced kidney function: A cross-sectional study using Japanese health examination data. Intern. Med..

[36] Zhu j., Shen L., Jia S., Wang W., Xiong Y. (2024). The role of uric acid in the risk of hypertension developed from prehypertension: A five-year Chinese urban cohort study. Arch. Public. Health.

[37] Wang R., WU S., Wang J., Li W., Cui J., Yao Z. (2024). A nonlinear correlation between the serum uric acid to creatinine ratio and the prevalence of hypertension: A large cross-sectional population-based study. Renal Fail..

[38] Xue T., Chen S., Wang P., Xu Q., Zhang X., Wu S., Luo Y., Wang A. (2023). Temporal relationship between hyperuricemia and hypertension and its impact on future risk of cardiovascular disease. Eur. J. Intern. Med..

[39] Mao T., He Q., Yang J., Jia L., Xu G. (2024). Relationship between gout, hyperuricemia, and obesity-does central obesity play a significant role?-a study based on the NHANES database. Diabetol. Metab. Syndr..

[40] Gong M., Wen S., Nguyen T., Wang C., Jin J., Zhou L. (2020). Converging relationships of obesity and hyperuricemia with special reference to metabolic disorders and plausible therapeutic implications. Diabetes Metab. Syndr. Obes..

[41] Battelli M.G., Bortolotti M., Polito L., Bolognesi A. (2018). The role of xanthine oxidoreductase and uric acid in metabolic syndrome. Biochim. Biophys. Acta Mol. Basis Dis..

[42] Hille R., Hall J., Basu P. (2014). The mononuclear molybdenum enzymes. Chem. Rev..

[43] Le Y., Zhou X., Zheng J., Yu F., Tang Y., Yang Z., Ding G., Chen Y. (2020). Anti-hyperuricemic effects of astaxanthin by regulating xanthine oxidase, adenosine deaminase and urate transporters in rats. Mar. Drugs.

[44] Dong Z., Zhou J., Jiang S., Li Y., Zhao D., Yang C., Ma Y., Wang Y., He H., Ji H. (2017). Effects of multiple genetic loci on the pathogenesis from serum urate to gout. Sci. Rep..

[45] Mandal A.K., Mount D.B. (2015). The molecular physiology of uric acid homeostasis. Annu. Rev. Physiol..

[46] Wen S., Arakawa H., Tamai I. (2024). Uric acid in health and disease: From physiological functions to pathogenic mechanisms. Pharmacol. Ther..

[47] Horsfall L.J., Irwin N., Irene P. (2014). Serum uric acid and the risk of respiratory disease: A population-based cohort study. Thorax.

[48] Su J., Wei Y., Liu M., Liu T., Li J., Ji Y., Liang J. (2014). Anti-hyperuricemic and nephroprotective effects of rhizoma Dioscoreae septemlobae extracts and its main component dioscin via regulation of mOAT1, mURAT1 and mOCT2 in hypertensive mice. Arch. Pharm. Res..

[49] Khanna D., Fitzgerald J.D., Khanna P.P., Bae S., Singh M.K., Neogi T., Pillinger M.H., Joan M., Shraddha P., Marian K. (2012). American College of Rheumatology guidelines for management of gout. Part 1: Systematic nonpharmacologic and pharmacologic therapeutic approaches to hyperuricemia. Arthritis Care Res..

[50] Oikawa M., Nishiwaki H., Hasegawa T., Sasaki S., Yazawa M., Miyazato H., Saka Y., Shimizu H., Fujita Y., Murakami M. (2024). The association between high-dose allopurinol and erythropoietin hyporesponsiveness in advanced chronic kidney disease: JOINT-KD study. Nephron.

[51] Jordan A., Gresser U. (2018). Side effects and interactions of the xanthine oxidase inhibitor febuxostat. Pharmaceuticals.

[52] Azevedo V.F., Kos I.A., Vargas-Santos A.B., da Rocha Castelar Pinheiro G., Dos Santos Paiva E. (2019). Benzbromarone in the treatment of gout. Adv. Rheumatol..

[53] Zhao Z., Liu J., Yuan L., Yang Z., Kuang P., Liao H., Luo J., Feng H., Zheng F., Chen Y. (2022). Discovery of novel benzbromarone analogs with improved pharmacokinetics and benign toxicity profiles as antihyperuricemic agents. Eur. J. Med. Chem..

[54] Francisca S., Mariano A., Nicola D. (2022). A glance into the future of gout. Ther. Adv. Musculoskelet. Dis..

[55] Dalbeth N., Phipps-Green A., Frampton C., Neogi T., Taylor W.J., Merriman T.R. (2018). Relationship between serum urate concentration and clinically evident incident gout: An individual participant data analysis. Ann. Rheum. Dis..

[56] Mao D., Feng J., Zhou Y., Li H. (2023). Analysis of different plant- and animal-based dietary patterns and their relationship with serum uric acid levels in Chinese adults. Nutr. J..

[57] Sarmadi B.H., Ismail A. (2010). Antioxidative peptides from food proteins: A review. Peptides.

[58] Lau J.L., Dunn M.K. (2018). Therapeutic peptides: Historical perspectives, current development trends, and future directions. Bioorg. Med. Chem..

[59] Li-Chan E.C. (2015). Bioactive peptides and protein hydrolysates: Research trends and challenges for application as nutraceuticals and functional food ingredients. Curr. Opin. Food. Sci..

[60] Du Z., Comer J., Li Y. (2023). Bioinformatics approaches to discovering food-derived bioactive peptides: Reviews and perspectives. TrAC Trends Anal. Chem..

[61] Onuh J.O., Aluko R.E. (2019). Metabolomics as a tool to study the mechanism of action of bioactive protein hydrolysates and peptides: A review of current literature. Trends Food Sci. Technol..

[62] Kubomura D., Yamada M., Masui A. (2016). Tuna extract reduces serum uric acid in gout-free subjects with insignificantly high serum uric acid: A randomized controlled trial. Biomed. Rep..

[63] Huang Y., Fan S., Lu G., Sun N., Wang R., Lu C., Han J., Zhou J., Li Y., Ming T. (2021). Systematic investigation of the amino acid profiles that are correlated with xanthine oxidase inhibitory activity: Effects, mechanism and applications in protein source screening. Free Radic. Biol. Med..

[64] Li W., Chen H., Chen H., Li Z., Hu W., Zhou Q., Xu B., Wang Y., Xing X. (2024). Andrias davidianus bone peptides alleviates hyperuricemia-induced kidney damage in vitro and in vivo. Food Sci. Hum. Wellness.

[65] Hao L., Ding Y., Fan Y., Xia C., Meng Y., Jia Q., Zhang J., Xue C., Hou H. (2024). New insights into anti-hyperuricemic effects of novel peptides from Antarctic Krill (*Euphausia superba*) by Q-Exactive Orbitrap MS-based non-targeted metabolomics. Food Biosci..

[66] Sun T., Li Y., Zhou J., Zhang D., Huang Z., He S., Li Y., Zhang C., Su X. (2019). Studies on the Effect of Apostichopus joponicus hydrolysate on the activity of xanthine oxidase. J. Chin. Inst. Food Sci. Technol..

[67] Murota I., Taguchi S., Sato N., Park E.Y., Nakamura Y., Sato K. (2014). Identification of antihyperuricemic peptides in the proteolytic digest of shark cartilage water extract using in vivo activity-guided fractionation. J. Agric. Food Chem..

[68] Bao C. (2014). Studies of Inhibitory Capability of Extracts from Uiva Pertusa and Gracilaria verrucosa Against Xanthine Oxidase and Anti-hyperuricemia in Mice. Master’s Thesis.

[69] Suwareh O., Causeur D., Le Feunteun S., Jardin J., Briard-Bion V., Pezennec S., Nau F. (2024). Proteins and bioactive peptides from algae: Insights into antioxidant, antihypertensive, antidiabetic and anticancer activities. Trends Food Sci. Technol..

[70] He W., Su G., Sun-Waterhouse D., Waterhouse G.I.N., Zhao M., Liu Y. (2019). In vivo anti-hyperuricemic and xanthine oxidase inhibitory properties of tuna protein hydrolysates and its isolated fractions. Food Chem..

[71] Zou L. (2019). Enzymatic Preparation and Functional Evaluation of Xanthine Oxidase Inhibitory Peptides from Skipjack Tuna. Master’s Thesis.

[72] Wei L., Ji H., Song W., Peng S., Zhan S., Qu Y., Chen M., Zhang D., Liu S. (2021). Hypouricemic, hepatoprotective and nephroprotective roles of oligopeptides derived from Auxis thazard protein in hyperuricemic mice. Food Funct..

[73] Zhan S. (2022). Preparation of XOD Inhibitory Peptides from *Sardinops sagax* and Their Uric Acid-Lowering Effects. Master’s Thesis.

[74] Sheng Z. (2018). Study on Uric Acid-Reducing Peptide of Tilapia Skin Collagen. Master’s Thesis.

[75] Song M. (2020). Screening of Xanthine Oxidase Inhibitor Peptides from Scomberomorus Niphonius Protein Based on Ligand Fishing. Master’s Thesis.

[76] Chen X., Guan W., Li Y., Zhang J., Cai L. (2023). Xanthine oxidase Inhibitory Peptides from *Larimichthys polyactis*: Characterization and in vitro/in silico evidence. Foods.

[77] Cui F., Xi L., Zhao G., Wang D., Tan X., Li J., Li T. (2022). Screening of xanthine oxidase inhibitory peptides by ligand fishing and molecular docking technology. Food Biosci..

[78] Zhao G., Li T., Song M., Sun H., Li J., Xie J., Deng S. (2021). Screening of xanthine oxidase inhibitory peptide from bass myosin by molecular docking. J. Chin. Inst. Food Sci. Technol..

[79] Yu Z., Kan R., Wu S., Guo H., Zhao W., Ding L., Zheng F., Liu J. (2021). Xanthine oxidase inhibitory peptides derived from tuna protein: Virtual screening, inhibitory activity, and molecular mechanisms. J. Sci. Food Agric..

[80] Li Y., Kang X., Li Q., Shi C., Lian Y., Yuan E., Zhou M., Ren J. (2018). Anti-hyperuricemic peptides derived from bonito hydrolysates based on in vivo hyperuricemic model and in vitro xanthine oxidase inhibitory activity. Peptides.

[81] Hu X., Zhou Y., Zhou S., Chen S., Wu Y., Li L., Yang X. (2021). Purification and Identification of Novel xanthine oxidase Inhibitory Peptides Derived from Round scad (*Decapterus maruadsi*) protein hydrolysates. Mar. Drugs.

[82] Hou M., Xiang H., Hu X., Chen S., Wu Y., Xu J., Yang X. (2022). Novel potential XOD inhibitory peptides derived from Trachinotus ovatus: Isolation, identification and structure-function analysis. Food Biosci..

[83] Bu Y., Wang F., Zhu W., Li X. (2020). Combining bioinformatic prediction and assay experiment to identify novel xanthine oxidase inhibitory peptides from Pacific bluefin tuna (*Thunnus orientalis*). E3S Web Conf..

[84] Zhao Q., Meng Y., Liu J., Hu Z., Du Y., Sun J., Mao X. (2022). Separation, identification and docking analysis of xanthine oxidase inhibitory peptides from pacific cod bone-flesh mixture. LWT.

[85] Zhao Q., Jiang X., Mao Z., Zhang J., Sun J., Mao X. (2023). Exploration, sequence optimization and mechanism analysis of novel xanthine oxidase inhibitory peptide from *Ostrea rivularis Gould*. Food Chem..

[86] Mao Z., Jiang H., Sun J., Mao X. (2023). Virtual screening and structure optimization of xanthine oxidase inhibitory peptides from whole protein sequences of Pacific white shrimp via molecular docking. Food Chem..

[87] Fan S., Huang Y., Lu G., Sun N., Wang R., Lu C., Ding L., Han J., Zhou J., Li Y. (2022). Novel anti-hyperuricemic hexapeptides derived from *Apostichopus japonicus* hydrolysate and their modulation effects on the gut microbiota and host microRNA profile. Food Funct..

[88] Kim S.K., WijeseKara I. (2010). Development and biological activities of marine-derived bioactive peptides: A review. J. Funct. Foods.

[89] Valencia P., Valdivia S., Nuñez S., Ovissipour R., Pinto M., Ramirez C., Perez A., Ruz M., Garcia P., Jimenez P. (2021). Assessing the enzymatic hydrolysis of salmon frame proteins through different by-product/water ratios and pH regimes. Foods.

[90] Zhao Q., Li Z., Liu Z., Zhao X., Fan Y., Dong P., Hou H. (2024). Preparation, typical structural characteristics and relieving effects on osteoarthritis of squid cartilage type II collagen peptides. Food Res. Int..

[91] Elango J., Robinson J., Zhang J., Bao B., Ma N., de Val J.E.M.S., Wu W. (2019). Collagen peptide upregulates osteoblastogenesis from bone marrow mesenchymal stem cells through MAPK-Runx2. Cells.

[92] Liu Y. (2014). Study on Anti-Gout Mechanism of Peptides Prepared from Marin Fish. Master’s Thesis.

[93] Guo Y. (2022). Preparation, Inhibitory Activity and Molecular Docking Mechanism of Bonito Roe XOD Inhibitory Peptide. Master’s Thesis.

[94] Ashraf Z.U., Gani A., Shah A., Gani A., Punoo H.A. (2024). Ultrasonication assisted enzymatic hydrolysis for generation of pulses protein hydrolysate having antioxidant and ACE-inhibitory activity. Int. J. Biol. Macromol..

[95] Themelis T., Gotti R., Orlandini S., Gatti R. (2019). Quantitative amino acids profile of monofloral bee pollens by microwave hydrolysis and fluorimetric high performance liquid chromatography. J. Pharm. Biomed. Anal..

[96] Chawla R., Patil G.R., Singh A.K. (2011). High hydrostatic pressure technology in dairy processing: A review. J. Food Sci. Technol..

[97] Choi J.S., Jang D.B., Moon H.E., Roh M.K., Kim Y.D., Cho K.K., Choi I.S. (2017). Physiological properties of *Engraulis japonicus* muscle protein hydrolysates prepared by subcritical water hydrolysis. J. Environ. Biol..

[98] Minkiewicz P., Dziuba J., Darewicz M., Iwaniak A., Dziuba M., Nałeçz D. (2008). Food peptidomics. Food Technol. Biotechnol..

[99] Javidfar M., Ahmadi S. (2020). QSAR modelling of larvicidal phytocompounds against Aedes aegypti using index of ideality of correlation. SAR QSAR Environ. Res..

[100] Deokar H., Deokar M., Wang W., Zhang R., Buolamwini J.K. (2018). QSAR studies of new pyrido[3,4-b]indole derivatives as inhibitors of colon and pancreatic cancer cell proliferation. Med. Chem. Res..

[101] Kumar N., Kaur K., Bedi P.M.S. (2023). Hybridization of molecular docking studies with machine learning based QSAR model for prediction of xanthine oxidase activity. Comp. Theor. Chem..

[102] Yong T., Xie Y., Chen S., Chen D., Su J., Jiao C., Hu H., Xiao C. (2018). Hypouricemic effect of *Grifola frondosa* on hyperuricemic mice and virtual screening of bioactives by 3D QSAR pharmacophore modeling. J. Funct. Foods.

[103] Meng P., Wang Y., Huang Y., Liu T., Ma M., Han J., Su X., Li W., Wang Y., Lu C. (2024). A strategy to boost xanthine oxidase and angiotensin converting enzyme inhibitory activities of peptides via molecular docking and module substitution. Food Chem..

[104] Bechaux J., Gatellier P., Le Page J.F., Drillet Y., Sante-Lhoutellier V. (2019). A comprehensive review of bioactive peptides obtained from animal byproducts and their applications. Food Funct..

[105] Wang X. (2015). Study on Isolation, Identification and Structure-Activity Relationship of the Constituents with Xanthine Oxidase Inhibitory Activities in Walnut *(Juglans regia* L.) Shell. Ph.D. Dissertation.

[106] Ma F., Sun S., Ye H., Zhang Z., Chen Q., Yin S., Cao Y., Miao J. (2024). Purification, characterization and anti-hyperuricemic mechanism of novel xanthine oxidase inhibitory peptides from tea (*Camellia sinensis* L.) protein. Food Biosci..

[107] Ma L., Hu J., Li J., Yang Y., Zhang L., Zou L., Gao R., Peng C., Wang Y., Luo T. (2018). Bisphenol A promotes hyperuricemia via activating xanthine oxidase. FASEB J..

[108] Li F., Liu Y., Xie Y., Liu Z., Zou G. (2020). Epigallocatechin gallate reduces uric acid levels by regulating xanthine oxidase activity and uric acid excretion in vitro and in vivo. Ann. Palliat. Med..

[109] Sun Z.R., Liu H.R., Hu D., Fan M.S., Wang M.Y., An M.F., Zhao Y.L., Xiang Z.M., Sheng J. (2021). Ellagic acid exerts beneficial effects on hyperuricemia by inhibiting xanthine oxidase and NLRP3 inflammasome activation. J. Agric. Food Chem..

[110] Dong Y., Sun N., Ge Q., Lv R., Lin S. (2023). Antioxidant soy peptide can inhibit xanthine oxidase activity and improve LO_2_ cell damage. Food Biosci..

[111] Chen Y., Lei H., Cao Z., Zhang C., Liu L., Gao X., Qin Q., Zhang L., Chen G. (2024). In vivo and in vitro insights into the anti-hyperuricemic effects of Sacha inchi (*Plukenetia volubilis* L.) leaves extract rich in polyphenols. Food Biosci..

[112] Hou C., Liu D., Wang M., Gong C., Li Y., Yang L., Yao M., Yuan E., Ren J. (2019). Novel xanthine oxidase-based cell model using HK-2 cell for screening antihyperuricemic functional compounds. Free Radic. Biol. Med..

[113] Wng Q., Xu C., Gu L. (2018). Fructose Induces HK-2 Cells to Express Monocyte Chemoattractant Protein-1 Through Uric Acid and Reactive Oxygen Species. J. Shanghai Jiaotong Univ. (Med. Sci.).

[114] Bjornstad P., Lanaspa M.A., Ishimoto T., Kosugi T., Kume S., Jalal D., Maahs D.M., Snell-Bergeon J.K., Johnson R.J., Nakagawa T. (2015). Fructose and uric acid in diabetic nephropathy. Diabetologia.

[115] Lu J., Hou X., Yuan X., Cui L., Liu Z., Li X., Ma L., Cheng X., Xin Y., Wang C. (2018). Knockout of the urate oxidase gene provides a stable mouse model of hyperuricemia associated with metabolic disorders. Kidney Int..

[116] Wu X., Wakamiya M., Vaishnav S., Geske R., Montgomery C., Jones P., Bradley A., Caskey C.T. (1994). Hyperuricemia and urate nephropathy in urate oxidase-deficient mice. Proc. Natl. Acad. Sci. USA.

[117] Pang L., Liang N., Li C., Merriman T.R., Zhang H., Yan F., Sun W., Li R., Xue X., Liu Z. (2024). A stable liver-specific urate oxidase gene knockout hyperuricemia mouse model finds activated hepatic de novo purine biosynthesis and urate nephropathy. Biochim. Biophys. Acta Mol. Basis Dis..

[118] Preitner F., Bonny O., Laverrière A., Rotman S., Firsov D., Da Costa A., Metref S., Thorens B. (2009). Glut9 is a major regulator of urate homeostasis and its genetic inactivation induces hyperuricosuria and urate nephropathy. Proc. Natl. Acad. Sci. USA.

[119] Takada T., Ichida K., Matsuo H., Nakayama A., Murakami K., Yamanashi Y., Kasuga H., Shinomiya N., Suzuki H. (2014). ABCG2 dysfunction increases serum uric acid by decreased intestinal urate excretion. Nucleosides Nucleotides Nucleic Acids.

[120] Xu L., Le S. (2006). A preliminary study on the establishment of a mouse model of hyperuricemia. Chin. J. Comp. Med..

[121] Pan Z., Duan F., Wang Y., Zhang J., Wei X. (2008). Effect of extract of cortex Phellodendri and Atractylodes Lancea on hyperuricemia in mice. Lishizhen Med. Mater. Med. Res..

[122] Zhang D., Liu H., Luo P., Li Y. (2018). Production Inhibition and Excretion Promotion of urate by fucoidan from *Laminaria japonica* in Adenine-Induced hyperuricemic Mice. Mar. Drugs.

[123] Liu X., Zhang L., Wu D., Liu J., Li G., Zhang Z., Li J. (2024). Three dietary phenols from pickled radish improve uric acid metabolism disorder in hyperuricemia mice associated with the altered gut microbiota composition. Food Biosci..

[124] Li L., Lin Z., Zhang B., Wu J., Wang H., Wang X., Niu H., Zhu C. (2014). Effect of high fructose drinking water on uric acid level in rats and the underlying pathological mechanism. Chin. J. Clin. Nutr..

[125] Ma C.H., Kang L.L., Ren H.M., Zhang D.M., Kong L.D. (2015). Simiao pill ameliorates renal glomerular injury via increasing Sirt1 expression and suppressing NF-κB/NLRP3 inflammasome activation in high fructose-fed rats. J. Ethnopharmacol..

[126] Bakker P.J., Butter L.M., Kors L., Teske G.J.D., Aten J., Sutterwala F.S., Florquin S., Leemans J.C. (2014). Nlrp3 is a key modulator of diet-induced nephropathy and renal cholesterol accumulation. Kidney Int..

[127] Miao C., Dong K., Shen Y., Sun Y., Li W., Man C., Zhang Y., Zhao Q., Jiang Y. (2024). Mechanism of *Lacticaseibacillus rhamnosus* JY027 alleviating hyperuricemia in mice through gut-kidney axis. Food Biosci..

[128] Mazzali M., Hughes J., Kim Y.G., Jefferson J.A., Kang D.H., Gordon K.L., Lan H.Y., Kivlighn S., Johnson R.J. (2001). Elevated uric acid increases blood pressure in the rat by a novel crystal-independent mechanism. Hypertension.

[129] Li Y., Zhu X., Liu F., Peng W., Zhang L., Li J. (2022). Pharmacodynamic evaluation of the XOR inhibitor WN1703 in a model of chronic hyperuricemia in rats induced by yeast extract combined with potassium oxonate. Curr. Res Pharmacol. Drug Discov..

[130] Zhang N., Zhang B., Chen X., Zhang Y., Wang Y., Lu S., Zhang H., Chen Y., Jiang H., Zhou H. (2024). Effects and mechanisms of *Polygonati rhizoma* polysaccharide on potassium oxonate and hypoxanthine-induced hyperuricemia in mice. Int. J. Biol. Macromol..

[131] Thisse C., Zon L.I. (2002). Organogenesis—Heart and blood formation from the zebrafish point of view. Science.

[132] Paw B.H., Zon L.I. (2000). Zebrafish: A genetic approach in studying hematopoiesis. Curr. Opin. Hematol..

[133] Nasevicius A.N.S., Ekker S.C. (2000). Effective targeted gene ‘knockdown’ in zebrafish. Nat. Genet..

[134] Chu J., Sadler K.C. (2009). New school in liver development: Lessons from zebrafish. Hepatology.

[135] Zhang Y., Li Q., Wang F., Xing C. (2019). A zebrafish (*Danio rerio*) model for high-throughput screening food and drugs with uric acid-lowering activity. Biochem. Biophys. Res. Commun..

[136] Han J., Wang X., Tang S., Lu C., Wan H., Zhou J., Li Y., Ming T., Wang Z.J., Su X. (2020). Protective effects of tuna meat oligopeptides (TMOP) supplementation on hyperuricemia and associated renal inflammation mediated by gut microbiota. FASEB J..

[137] Nongonierma A.B., FitzGerald R.J. (2012). Tryptophan-containing milk protein-derived dipeptides inhibit xanthine oxidase. Peptides.

[138] Nongonierma A.B., Mooney C., Shields D.C., Fitzgerald R.J. (2013). Inhibition of dipeptidyl peptidase IV and xanthine oxidase by amino acids and dipeptides. Food Chem..

[139] Adamson N.J., Reynolds E.C. (1996). Characterization of casein phosphopeptides prepared using alcalase: Determination of enzyme specificity. Enzyme Microb. Technol..

[140] Tacias-Pascacio V.G., Morellon-Sterling R., Siar E.H., Tavano O., Berenguer-Murcia Á., Fernandez-Lafuente R. (2020). Use of alcalase in the production of bioactive peptides: A review. Int. J. Biol. Macromol..

[141] Li Q. (2018). Study on the Structure–Activity Mechanism of Targeting Inhibition of Xanthine Oxidase by Uric Acid-Lowering Peptides Derived from Walnut. Ph.D. Dissertation.

[142] Hou M., Hu X., Yang X., Chen S., Wu Y., Xu J. (2021). Preparation and process optimization of xanthine oxidase inhibitory peptides from *Trachinotus ovatus*. Food Ferment. Ind..

[143] Klomklao S., Kishimura H., Yabe M., Benjakul S. (2007). Purification and characterization of two pepsins from the stomach of pectoral rattail (*Coryphaenoides pectoralis*). Comp. Biochem. Physiol. B Biochem. Mol. Biol..

[144] Tejpal C.S., Vijayagopal P., Elavarasan K., Linga Prabu D., Lekshmi R.G.K., Asha K.K., Anandan R., Chatterjee N.S., Mathew S. (2017). Antioxidant, functional properties and amino acid composition of pepsin-derived protein hydrolysates from whole tilapia waste as influenced by pre-processing ice storage. J. Food Sci. Technol..

[145] Granger D.N. (1988). Role of xanthine oxidase and granulocytes in ischemia-reperfusion injury. Am. J. Physiol..

[146] Chu Y., Sun J., Zhu Q., Yao W., Song R., Wang J. (2023). Optimization of preparation and in vitro activity of uricate-lowering peptide from dorsal belly meat of skipjack. Food Mach..

[147] Lassoued I., Mora L., Nasri R., Aydi M., Toldrá F., Aristoy M.C., Barkia A., Nasri M. (2015). Characterization, antioxidative and ACE inhibitory properties of hydrolysates obtained from thornback ray (*Raja clavata*) muscle. J. Proteom..

[148] Wu R., Wu C., Liu D., Yang X., Huang J., Zhang J., Liao B., He H. (2018). Antioxidant and anti-freezing peptides from salmon collagen hydrolysate prepared by bacterial extracellular protease. Food Chem..

[149] Wu R., Wu C., Liu D., Yang X., Huang J., Zhang J., Liao B., He H., Li H. (2015). Overview of antioxidant peptides derived from marine resources: The sources, characteristic, purification, and evaluation methods. Appl. Biochem. Biotechnol..

[150] Ping F. (2016). Research on the Mechanism of Uric Acid Nephropathy Interfered by Bixiechubi Decoction Regulating NLRP3 Inflammasome Through PI3K/AKT/mTOR Pathway. Ph.D. Thesis.

[151] Yu S., Ren Q., Wu W. (2015). Effects of losartan on expression of monocyte chemoattractant protein-1 (MCP-1) in hyperuricemic nephropathy rats. J. Recept. Signal Transduct. Res..

[152] Li L., Yang C., Zhao Y., Zeng X., Liu F., Fu P. (2014). Is hyperuricemia an independent risk factor for new-onset chronic kidney disease?: A systematic review and meta-analysis based on observational cohort studies. BMC Nephrol..

[153] Yang C.S., Shin D.M., Jo E.K. (2012). The role of NLR-related Protein 3 inflammasome in host defense and inflammatory diseases. Int. Neurourol. J..

[154] Deng Y., Han H., He L., Deng D., Wang J., Yin Y., Li T. (2022). Effects of Lysine–Lysine Dipeptide on Serum Amino Acid Profiles, Intestinal Morphology, and Microbiome in Suckling Piglets. Front. Nutr..

[155] Clemente A., Vioque J., Sánchez-Vioque R., Pedroche J., Bautista J., Millán F. (1999). Protein quality of chickpea (*Cicer arietinum* L.) protein hydrolysates. Food Chem..

[156] Abeer M.M., Trajkovic S., Brayden D.J. (2021). Measuring the oral bioavailability of protein hydrolysates derived from food sources: A critical review of current bioassays. Biomed. Pharmacother..

[157] Pasukamonset P., Kwon O., Adisakwattana S. (2016). Alginate-based encapsulation of polyphenols from Clitoria ternatea petal flower extract enhances stability and biological activity under simulated gastrointestinal conditions. Food Hydrocoll..

[158] Yi H., Yu H., Wang L., Wang Y., Ouyang C., Keshta B.E. (2024). Microneedle transdermal drug delivery as a candidate for the treatment of gouty arthritis: Material structure, design strategies and prospects. Acta Biomater..

[159] Li D., Dong J., Xiong T., Zhou X., Li Y., Chen C., Li S., Song Z., Xu N., Yang M. (2024). Transdermal delivery of iguratimod and colchicine ethosome by dissolving microneedle patch for the treatment of recurrent gout. Colloids Surf. B Biointerfaces.

